# Rational Design of Novel Peptidomimetics against Influenza A Virus: Biological and Computational Studies

**DOI:** 10.3390/ijms241814268

**Published:** 2023-09-19

**Authors:** Maria Carmina Scala, Magda Marchetti, Fabiana Superti, Mariangela Agamennone, Pietro Campiglia, Marina Sala

**Affiliations:** 1 Department of Pharmacy, University of Salerno, Via Giovanni Paolo II 132, 84084 Fisciano, Italy; mscala@unisa.it (M.C.S.); pcampiglia@unisa.it (P.C.); 2 National Centre for Innovative Technologies in Public Health, National Institute of Health, Viale Regina Elena, 299, 00161 Rome, Italy; magda.marchetti@iss.it (M.M.); or fabiana.superti@artoi.it (F.S.); 3 Association for Research on Integrative Oncology Therapies (ARTOI) Foundation, Via Ludovico Micara, 73, 00165 Rome, Italy; 4 Department of Pharmacy, University “G. d’Annunzio” of Chieti-Pescara, Via dei Vestini 31, 66100 Chieti, Italy; magamennone@unich.it

**Keywords:** influenza, hemagglutinin, peptidomimetic, biophysical assay, docking

## Abstract

Effective therapy against the influenza virus is still an unmet goal. Drugs with antiviral effects exist, but the appearance of resistant viruses pushes towards the discovery of drugs with different mechanisms of action. New anti-influenza molecules should target a good candidate, as a new anti-influenza molecule could be an inhibitor of the influenza A virus hemagglutinin (HA), which plays a key role during the early phases of infection. In previous work, we identified two tetrapeptide sequences, SLDC (**1**) and SKHS (**2**), derived from bovine lactoferrin (bLf) C-lobe fragment 418–429, which were able to bind HA and inhibit cell infection at picomolar concentration. Considering the above, the aim of this study was to synthesize a new library of peptidomimetics active against the influenza virus. In order to test their ability to bind HA, we carried out a preliminary screening using biophysical assays such as surface plasmon resonance (SPR) and orthogonal immobilization-free microscale thermophoresis (MST). Biological and computational studies on the most interesting compounds were carried out. The methods applied allowed for the identification of a N-methyl peptide, S(N-Me)LDC, which, through high affinity binding of influenza virus hemagglutinin, was able to inhibit virus-induced hemagglutination and cell infection at picomolar concentration. This small sequence, with high activity, represents a good starting point for the design of new peptidomimetics and small molecules.

## 1. Introduction

Influenza A virus (IAV) is an extremely infectious pathogen endowed with zoonotic potential and represents one of the leading causes of morbidity and mortality worldwide [1,2]. Over the decades, it has triggered several pandemics, causing major public health problems as well as severe economic losses [3,4]. Flu therapy is based on vaccine prevention and antiviral drug treatment. However, vaccines have several limitations. They are not always protective against the circulating strains, and they cannot be administered to immunosuppressed patients or, in any case, to those with a weak immune system.

Antivirals are an alternative approach to combating the various subtypes of influenza viruses [5]. Antiviral compounds approved by the Food and Drug Administration (FDA) for flu treatment can be classified into three groups: the neuroaminidase (NA) inhibitors oseltamivir phosphate, zanamivir, peramivir, and laninamivir octanoate hydrate; the M2 ion channel blockers amantadine and rimantadine; and the cap-dependent endonuclease protein inhibitor baloxavir marboxil [6,7,8,9,10]. However, the emerging threat of new pandemic influenza strains spreading through the human population and the rise of resistance to licensed drugs have focused research into developing new treatments for influenza viruses [11,12,13,14]. A viral component whose role in virus infection is crucial should be the ideal target for novel anti-influenza treatments.

The IAV envelope contains two glycoproteins: hemagglutinin (HA) and neuraminidase (NA). Both play pivotal roles in the infection cycle: HA for binding to specific cell receptors and mediating membrane fusion, and NA for releasing viral particles from the cell surface, but they are also involved in viral attachment and entry. The majority of current treatment approaches for IAV target NA. Oseltamivir, the only oral NA inhibitor, is the most common anti-flu therapy worldwide, so its broad use led to the selection of resistant viral strains [15]. Frequently, when a tyrosine residue replaces the histidine residue 274 (position 275 of the N1 protein) on the NA gene, influenza A/H1N1 viruses develop oseltamivir resistance [16]. For example, during the 2007–2008 influenza season in Europe, many seasonal A(H1N1) viruses emerged with the H275Y NA mutation, conferring a high resistance to oseltamivir [17]. It is important to note that NA mutations responsible for oseltamivir resistance can emerge independently of the use of this drug [17]. In fact, a high proportion of oseltamivir-resistant viruses have been isolated from patients who have not received treatment, demonstrating a high rate of transmission [6]. Moreover, as these mutants are known to maintain fitness, they can easily spread globally, representing a serious public health risk. Furthermore, it is important to note that potential pandemic viruses, such as avian influenza A(H5N1) and A(H7N9), which cause severe disease in humans, are prone to the development of oseltamivir resistance in treated patients [18,19,20]. Finally, it has been demonstrated that mallards can transmit an avian influenza virus that is oseltamivir-resistant to chickens. As this mutant is able to cross the species barrier and also maintain fitness, it could be able to infect humans [21]. Finding alternative antiviral drugs with distinct modes of action is therefore urgently needed to fight infection by emerging viruses with pandemic potential, especially those carrying drug resistance markers. However, attempts to target HA are on the rise. The discovery of broadly neutralizing antibodies that target the HA stem has led to increased attention for HA as a target for broadly cross-protective antivirals. These antibodies are under investigation for direct use as therapeutic agents and have inspired the design of smaller cyclic peptides based on the antibody-binding interface.

Previously, Superti and coworkers demonstrated that bLf, in particular the bLf C-lobe, can bind and inhibit HA [22]. Successively, through a truncation library, we identified the tetrapeptides SLDC (**1**) and SKHS (**2**), derived from bLf C-lobe fragment 418–429, which were able to bind HA and inhibit cell infection in a concentration range of picomolar [23].

It is well known that the direct application of peptides as medicinal entities has some severe limitations, including high degradation by proteolytic enzymes and poor cell membrane permeability. We designed new peptidomimetics derived from tetrapeptides **1** and **2** to increase metabolic stability and improve bioavailability, receptor affinity, and selectivity to overcome these drawbacks. Biological and computational assays on synthesized compounds were carried out. Direct binding assays, a bioactivity profile, and computational studies led to the identification of a very potent N-methyl peptide.

## 2. Results and Discussion

### 2.1. Design

Novel peptidomimetics were designed starting from the tetrapeptide sequences SLDC (**1**) and SKHS (**2**). Two different strategies were used to reduce the conformational freedom of the peptide backbone and increase the affinity towards the target: N-methylation and the synthesis of peptoids.

#### 2.1.1. Design of N-Methyl Peptides

In peptide chemistry, N-methylation has been demonstrated to be a useful technique in structure–activity relationship studies. In physiologically active peptides, substitution of natural amino acids with N-methyl amino acids has enabled analogs with better pharmacological properties, such as enzymatic stability [24,25]**,** receptor selectivity [26,27], enhanced potency [28,29,30]**,** and bioavailability [31,32,33,34]. In fact, N-methylation of the peptide backbone gives peptides high affinity for their targets, proteolytic stability, membrane permeability, and conformational rigidity.

Hence, starting with tetrapeptides **1** and **2**, we synthesized the corresponding N-methyl peptides (**3**–**10**, Table 1). Therefore, we exchanged in a systematic manner Ser, Leu, Asp, Cys, Lys, and His with their N^α^-methylated analogs.

#### 2.1.2. Design of Peptoids

**Figure 1:**
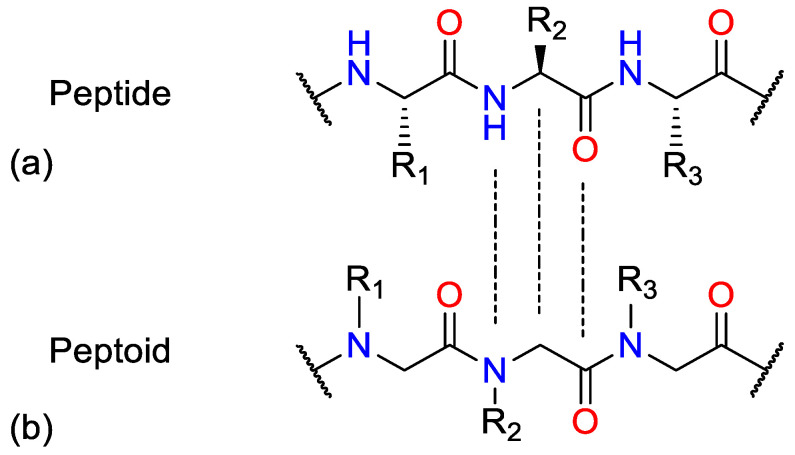
Comparison of (**a**) a peptide and (**b**) a peptoid.

Repeated combinations of poly-N-substituted glycine (NSG) form peptidomimetic compounds known as peptoids [35]. They are a class of artificial, unnatural peptide mimics that have a modular structure that allows a wide variety of structural components to be easily incorporated. Peptoids have the functionality of their side chains linked to the nitrogen of the polyamide backbone instead of the α-carbon, creating an achiral and flexible oligomeric backbone without hydrogen bond donors. This N-alkyl backbone repeat motif allows peptoids greater stability against proteolytic degradation than analogous peptides [36]. Additionally, NSGs may be more hydrophobic and possess higher cellular permeability [37,38,39]. Figure 1 shows the similarities between peptides and peptoids in the spacing of the side chains and carbonyl groups and the variations in the chirality of the two monomers [40].

Hence, we synthesized the peptoid analogues of **1** and **2** (**11**–**18**), replacing in a systematic manner Ser, Leu, Asp, Cys, Lys, and His with their NGS.

### 2.2. Chemistry

#### 2.2.1. N-Methyl Peptides

**Scheme 1:**
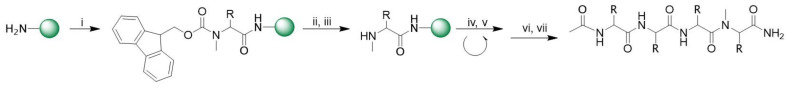
General synthesis of N-methylated peptide. Reagents and conditions: (i) Fmoc-N-Me-Ser(tBu)-OH (3 eq.), HBTU (3 eq.), HOAt (3 eq.), DIPEA (6 eq.), 2 × 20 min, MW 35 °C; (ii) piperidine/N,N-dimethylformamide (DMF, 2:8, *v*/*v*) × 10 min rt; (iii) piperidine/DBU/toluene/DMF (5:5:20:70, *v*/*v*) 2 × 5 min rt; (iv) Fmoc-L-aa(PG)-OH (3 eq.), HBTU (3 eq.), HOAt (3 eq.), DIPEA (6 eq.), 2 × 10 min, MW 75 °C; (v) 20% piperidine/DMF 1 × 30 min rt; (vi) Ac_2_O/ dichloromethane (DCM) (1:3 rt); (vii) TFA/TIS/H_2_O (95:2.5:2.5 *v*/*v*).

Over the decades, several methods for solid-phase synthesis of N-methylated peptides have been described [41,42]. The major limitation is the coupling of the N-methyl amino acid to the previous amino acid, which generally occurs in low yield and requires expensive coupling reagents and double coupling due to the sterically hindered N-methyl site. In this work, we planned to use N-methylated amino acids directly as building blocks, aided by microwave (MW) irradiation. Fmoc-Rink Amide Chem matrix resin was used as solid phase support (Scheme 1).

#### 2.2.2. Peptoids

In the solid-phase synthesis of peptoid, there are two distinct methods to introduce a *N*-alkylglycine (peptoid residue) on the growing peptide chain: (i) the N-substituted glycine derivative, appropriately protected at the tertiary nitrogen atom, can be prepared distinctly and used as a building block in the solid-phase procedure (monomer method) [43,44], or, alternatively, (ii) the peptoid residue is created during the growing peptide chain by combining two submonomers, a haloacetic acid and a primary amine (submonomer approach) [35].

We chose and improved the submonomer method, which allows direct assembly of the functionalized peptoid residue from commercially available chemicals, to speed up the synthesis of peptoids.

The peptoid residue is created during the peptide chain elongation for the synthesis of compounds **11**–**18** by mixing two submonomers, a haloacetic acid, and a primary amine (submonomer technique). Initially, we used a 25% piperidine solution in DMF for 30 min to deprotect the Rink Amide resin.

**Scheme 2:**
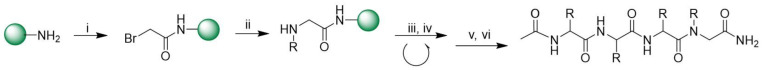
General synthesis of peptoids. Reagents and conditions: (i) bromoacetic acid/DMF (2M) and DIC (10 eq.), 10 min, MW 75 °C; (ii) R-NH_2_/DMF (1M), 10 min, MW 75 °C; (iii) piperidine/DMF (2:8, *v*/*v*) × 10 min rt; (iv) Fmoc-L-aa(PG)-OH (3 eq.), HBTU (3 eq.), HOAt (3 eq.), DIPEA (6 eq.), 2 × 10 min, MW 75 °C; (v) 20% piperidine/DMF 1 × 30 min rt; (vi) Ac_2_O/DCM (1:3 rt); (vii) TFA/TIS/H_2_O (95:2.5:2.5 *v*/*v*).

Bromoacetic acid was then coupled to the NH_2_-peptide resin in the presence of N, N′-diisopropylcarbodiimide (DIC), and the halogen was subsequently removed using an excessive amount of primary amine. Peptide chain elongation was carried out according to the standard protocol by adding the appropriate amine and bromoacetic acid. The last *N*-alkylglycine residue was acetylated as described in Section 3. Peptoids were simultaneously cleaved from the resin and deprotected. The synthesis is shown in Scheme 2.

The crude compounds were purified by preparative RP-HPLC. Analytical HPLC indicated a purity greater than 98%, and molecular weights were confirmed by ESI-MS.

### 2.3. Direct Binding Assays

#### 2.3.1. Microscale Thermophoresis (MST)

**Figure 2:**
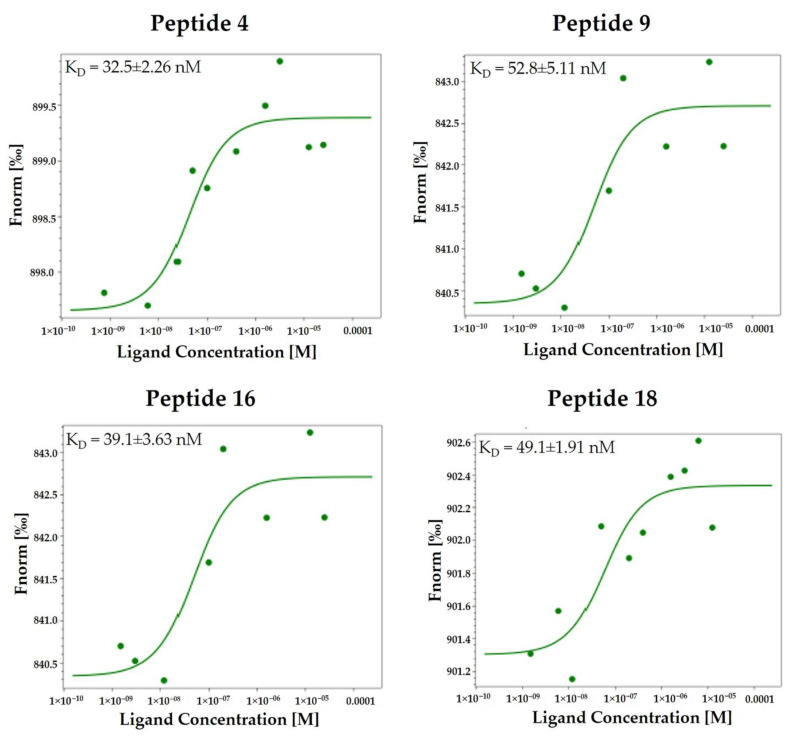
Direct binding measurements of the interaction between peptidomimetics **4**, **9**, **16,** and **18** and HA (H1N1 hemagglutinin, Cat No.: Z03181, GenScript, NE). MST binding affinity assays were carried out as described in the Materials and Methods section (Section 3). The single dose–response curves for the binding of the peptidomimetics **4**, **9**, **16**, and **18** to HA are depicted; F_norm_, normalized fluorescence. The experiments were repeated independently three times. The reported K_D_ is the mean SD of three independent experiments.

The 16 compounds were subjected to MST screening, as described in Materials and Methods (Section 3), and the results are shown in Table 1. According to the analysis, every peptide interacts with HA with different dissociation constants. Compounds **4**, **9**, **16**, and **18** bind HA more effectively than compounds **1** and **2**, showing an equilibrium dissociation constant (KD) value in a two-digit nanomolar concentration range (Figure 2).

#### 2.3.2. Surface Plasmon Resonance (SPR)

Full-length recombinant HA (His-Tag) was immobilized up to about 11,000 response units (RU) on a sensor chip for the SPR study (Materials and Methods, Section 3). On the sensor surface, compound binding caused a change in refractive index [45]. A regeneration step was required (glycine, pH 1.5). After injection, a running buffer was allowed to flow over the surface, and the dissociation of compounds from the surface was detected. On the other hand, no HA was immobilized on the control flow cell, so no appreciable signal alteration occurred.

**Figure 3:**
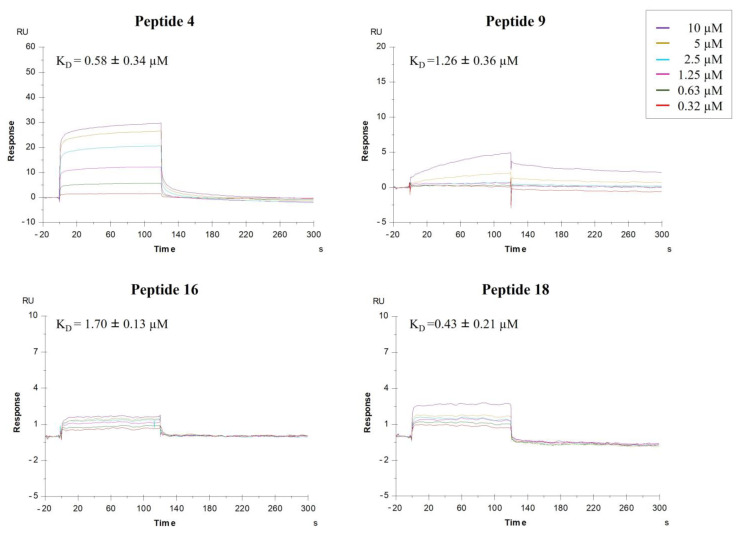
Surface plasmon resonance (SPR) analysis. Sensorgrams to evaluate the binding affinity between HA (H1N1 hemagglutinin, Cat No. Z03181, GenScript, NE) and peptidomimetics **4**, **9**, **16**, and **18**. Each compound was injected at six different concentrations (0.32, 0.63, 1.25, 2.5, 5, and 10 µM). The ratio between the kinetic dissociation (k_off_) and association (k_on_) constants was used to determine the equilibrium dissociation constants (K_D_).

The SPR study revealed that synthesized peptides effectively interact with the immobilized protein. The sensorgrams of the compounds **4**, **9**, **16**, and **18** bound to HA in HBS-P buffer are shown in Figure 3. Interestingly, these peptidomimetics bind HA with higher efficiency with respect to **1** and **2**, showing a K_D_ value in a three-digit nanomolar concentration range.

Specific binding between compounds **4**, **9**, **16**, and **18** and HA was shown by both direct binding assays.

### 2.4. Antiviral Activity

#### 2.4.1. Toxicity of Compounds in MDCK cells

Two-fold serial dilutions of selected compounds (starting at 25 µM) were incubated for 72 h at 37 °C with semi-confluent MDCK cells grown in 96-well tissue culture microplates. Under these conditions, no peptide affected cell viability up to the highest concentration.

#### 2.4.2. Hemagglutination Inhibition Assay (HI)

The capability of compounds **4**, **9**, **16**, and **18** to inhibit hemagglutinating activity was evaluated by HI. The inhibitory activity of peptides **1** and **2** is shown as a reference. The oseltamivir-resistant A/Parma/24/09 H1N1 virus strain was used. As shown in Table 2, compounds **4** and **16** prevented the hemagglutination of the oseltamivir-resistant strain A/Parma/24/09 H1N1 at quite similar concentrations to the reference peptides.

#### 2.4.3. Neutralization Assay (NT)

Next, we evaluated the ability of the four compounds to inhibit viral infection by neutralization assay. All compounds were able to prevent viral infection in a dose range of roughly 50 to 140 pM, corresponding to selectivity indices ≈ 10^3^/10^5^. In particular, compound 4 inhibited virus replication at a concentration similar to that of the reference peptide (Table 3).

### 2.5. Computational Studies

The computational analysis of synthesized and tested compounds follows the previously generated protocol [46]. Briefly, the hemagglutinin 3D structure was generated by homology modeling using the exact sequence of the assayed viral strains [46]. The Receptor Binding Site, responsible for recognizing the sialic acid and highlighted by the SiteMap analysis on the HA surface, was selected to perform the docking calculations. Structure-based studies exploited the SP-peptide protocol available in Glide to explore the large conformational flexibility of the studied ligands. As already described, the 100 final poses were clustered to obtain each ligand’s best representative binding geometry.

As all compounds showed activity toward A/Parma/24/09 H1N1, we focused our study on this viral strain’s hemagglutinin. Additionally, we report the discussion about the most interesting compounds (**4**, **9**, **16**, and **18**) binding to the RBS of A/Parma/24/09 H1N1. Docking score values for the four new ligands in comparison with previously studied tetrapeptides are reported in Table 4. The Glide calculated score is similar for all ligands and in line with the HI activity in Table 2.

**Figure 4:**
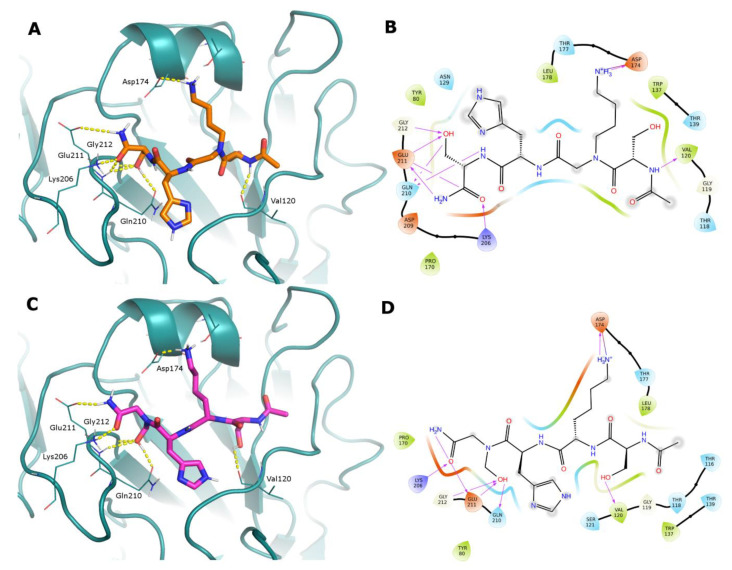
(Panels (**A**,**C**) Docked poses of peptoids **16** and **18** represented as sticks (orange and purple C atoms, respectively) in the RBS of A/Parma/24/09 H1N1 (deep-cyan cartoon). Just the HA residues that interact with the ligands are shown as lines for clarity’s sake. H-bonds are represented as yellow dashed lines. (Panels (**B**,**D**)) Corresponding 2D ligand interaction diagrams; in these diagrams, residues close to the ligand are colored according to their properties (orange, negatively charged; blue, positively charged; green, hydrophobic; cyan, polar); H-bonds are shown as magenta arrows; and a gray shadow encircles atoms that are exposed to the solvent.

Compounds **16** and **18** are SKHS peptoids where the side chains of Lys2 and Ser4 residues, respectively, are moved on the peptide nitrogen atom. The docked binding mode follows the same profile we observed previously for the lead tetrapeptide SKHS (Figure S17): the ligands occupy the water-exposed RBS in an extended conformation, with Ser4 forming a network of hydrogen bonds with Gln210, Glu211, and Gly212. Differently from SKHS, the lysine side chain is able to form an ionic contact with Asp174 in both cases (Figure 4).

**Figure 5:**
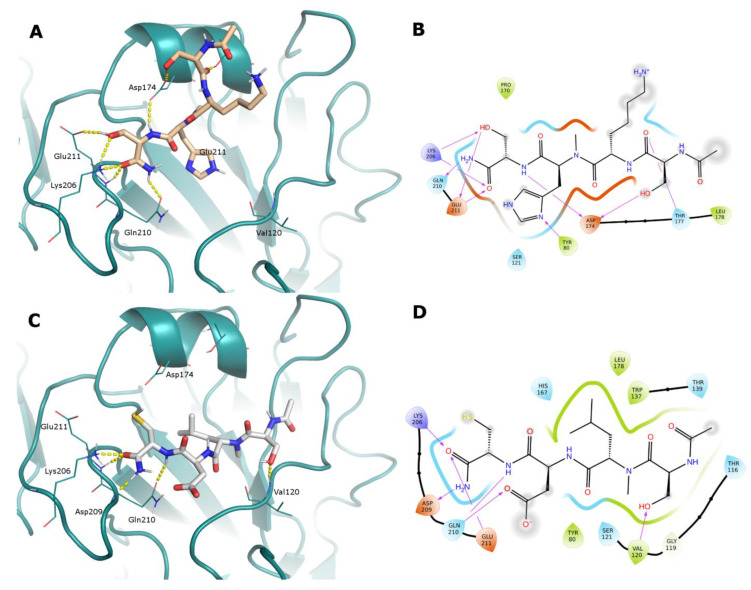
(Panels (**A**,**C**)) Docked poses of N-methyl peptides **9** and **4** represented as sticks (light orange and white C atoms, respectively) in the RBS of A/Parma/24/09 H1N1 (deep-cyan cartoon). Just the HA residues that interact with the ligands are shown as lines for clarity’s sake. H-bonds are represented as yellow dashed lines. (Panels (**B**,**D**)) Corresponding 2D ligand interaction diagrams; in these diagrams, residues close to the ligand are colored according to their properties (orange, negatively charged; blue, positively charged; green, hydrophobic; cyan, polar); H-bonds are shown as magenta arrows; and a gray shadow encircles atoms that are exposed to the solvent.

The N-methyl derivative **9** presents reduced conformational flexibility that hampers the contact between the Lys2 protonated amine and Asp174 and causes a different ligand arrangement in the binding site. In fact, while the Ser4 OH occupies its conserved position in contact with Glu211, the serine residue in the opposite position reaches Asp174 to form an H-bond. Other H-bonds are formed with Thr177, Tyr80, Gln210, Glu211, and Tyr206 (Figure 5A,B).

Compound **4** is the derivative of SLDC where the Leu2 NH is methylated. Its docked interactions are driven by the hydrophobic contact of the Leu2 side chain in the most lipophilic region of the RBS. To satisfy this contact, the ligand inverts its binding mode with respect to the lead SLDC (Figure S18). The cysteine residue backbone forms H-bonds with key residues Gln210, Lys206, and Asp208, while Ser1 OH interacts with Val120 (Figure 5C,D).

## 3. Materials and Methods

### 3.1. Peptide Synthesis

N^α^-Fmoc-protected amino acids, Fmoc-Rink Amide AM resin, 1-hydroxy-7-azabenzotriazole (HOAt), N-hydroxy-benzotriazole (HOBt), 2-(1H-benzotriazole-1-yl)- 1, 1, 3, 3-tetramethyluronium hexafluoro-phosphate (HBTU), N, N-diisopropylethyl-amine (DIPEA), piperidine, and trifluoroacetic acid were acquired from Iris Biotech (Marktredwitz, Germany). Biotage AB (Uppsala, Sweden) supplied the Rink Amide ChemMatrix resins. Unless otherwise stated, solvents and reagents for peptide synthesis, as well as CH_3_CN for high-performance liquid chromatography (HPLC), were of reagent grade and purchased from commercial sources.

#### 3.1.1. Synthesis of N-Methyl Peptides

After placing Fmoc-Rink Amide resin (100–200 mesh, 1% DVB, 0.75 mmol/g) in a peptide synthesis vessel, it was swelled in DMF and deprotected with a 20% piperidine/DMF solution (5 mL, 1 × 5 min and 1 × 25 min). Protected amino acid (3 eq.), HBTU (3 eq.), and HOBt (3 eq.) in DMF (1–3 mL/g resin) were sequentially added to the resin, and the mixture was irradiated in an automated microwave peptide synthesizer (Biotage Initiator + Alstra™) for 20 min with a maximum temperature of 35 °C.

The following protected N-methyl amino acids were then added stepwise: Fmoc-N-Me-Lys(Boc)-OH, Fmoc-N-Me-His(Trt)-OH, Fmoc-N-Me-Leu-OH, Fmoc-N-Me-Asp(OtBu)-OH, Fmoc-N-Me-Cys(Trt)-OH, Fmoc-N-Me-Ser(tBu)-OH.

The peptide resin was washed with DCM (3×), DMF (3×), and DCM (3×), and the Fmoc deprotection protocol described above was repeated after each coupling step.

Two treatments with piperidine/DMF (2:8, *v*/*v*) for 10 min and two extra 5 min treatments with piperidine/DBU/toluene/DMF (5:5:20:70, *v*/*v*) were used. Additionally, the chloranil test was carried out following each phase of deprotection and coupling to validate, respectively, the complete removal of the Fmoc protective group and to confirm that complete coupling has taken place on all the free amines on the resin. After removing the N-terminal Fmoc group, the peptides were acetylated by adding a solution of Ac_2_O/DCM (1:3) and agitating for 30 min. The final step was cleaving the peptides from the resin using the following cleavage cocktails: TFA/DCM (95:5 *v*/*v*) for 90 min (10 mL/g resin). By adding cold diethyl ether, peptides were precipitated, the solution was decanted, the solid was triturated, and the mixture was then again decanted. This procedure was carried out twice. The yield for the crude peptides was 30%.

#### 3.1.2. Synthesis of Peptoids

A 2 M solution of bromoacetic acid in DMF (10 eq.) and DIC (10 eq.) was applied to the deprotected Rink Amide resin (100–200 mesh, 1% DVB, 0.59 mmol/g) in order to assemble N^κ^-protected N-N-aminoalkyl glycine residue on-resin. A 1 M solution of the selected amine in DMF was added after washing in DMF. In the Biotage Initiator + Alstra automated microwave synthesizer, the acylation reactions and nucleophilic displacement were carried out.

Then, the protected amines listed below were gradually added: Tert-butyl 4-(aminomethyl)-1H-imidazole-1-carboxylate, N-Boc-1,4-butanediamine, 2-(tert-butoxy)ethan-1-amine, Glycine tert-butyl ester hydrochloride, 2-methyl-2-propanethiol, and 2-methylpropan-1-amine.

The beads were washed with DMF and prepared for the formation of the next residue. The finished peptoid resin was treated with a TFA-H_2_O-triisopropylsilane (TIS) mixture (95:2.5:2.5 by volume) for 90–120 min at rt to cleave the peptides from the resin and remove the acid-labile protecting groups. By adding cold diethyl ether, peptides were precipitated, then dried overnight under vacuum. A yield of 70–80% crude peptides was achieved.

#### 3.1.3. Purification and Characterization

All crude peptides were purified by RP-HPLC on a preparative C18-bonded silica column (Phenomenex Kinetex AXIA 100 Å, 100 × 21.20 mm, 5 µm) using a Shimadzu SPD 20A UV/VIS detector, with detection at 214 and 254 nm. The column was perfused at a flow rate of 15 mL/min with solvent A (5%, *v*/*v*, water in 0.1% aqueous TFA), and a linear gradient from 5 to 90% of solvent B (85%, *v*/*v*, acetonitrile in 0.1% aqueous TFA) over 20 min was adopted for peptide elution. Analytical purity and retention time (tr) of each peptide were determined using HPLC conditions in the above solvent system (solvents A and B) programmed at a flow rate of 1.500 mL/min using a linear gradient from 5 to 90% B over 8 min, fitted with a C-18 column (Phenomenex, Aeris XB-C18 column; 150 mm × 4.60, 3.6 µm). All analogs showed >97% purity when monitored at 215 nm. Homogeneous fractions, as established using analytical HPLC, were pooled and lyophilized. Peptide molecular weights were determined by positive ESI infusion on a Q-Tof Premier Mass Spectrometer (Waters), equipped with the Xcalibur software (v4.2.47) for processing the data acquired. The sample was dissolved in a mixture of water and methanol (50/50) and injected directly into the electrospray source using a syringe pump at a constant flow (15 μL/min). Analytical data are shown in the Supplementary Materials (Table S1 and Figures S1–S16).

### 3.2. Direct Binding Assay

HA was purchased from GenScript (Piscataway, NJ, USA), Cat No.: Z03181-100. CM5 sensor chips, HBS-P+ buffer (0.01 M HEPES pH 7.4, 0.15 M NaCl, 0.05% *v*/*v* Surfactant P20), 1-ethyl-3- (3-diaminopropyl) carbodiimide hydrochloride (EDC), N-hydroxysuccinimide (NHS), ethanolamine (NH_2_CH_2_CH_2_OH), and regeneration solution were purchased from Cytiva (Uppsala, Sweden).

#### 3.2.1. Microscale Thermophoresis (MST)

The Monolith NT.115pico (Nano-Temper Technologies GmbH, Munich, Germany) instrument was used for MST studies. His-HA was labeled using the Nanotemper His-Tag Labeling Kit RED-tris-NTA 2nd Generation as described elsewhere [47]. His-HA protein (100 µL, 80 nM) in ddH_2_O was added to NT647-NHS fluorophore (100 µL, 40 nM, NanoTemper Technologies GmbH, Munich, Germany) in labeling buffer and incubated for 30 min at rt. Protein aggregates were removed from NT647-HA by centrifuging it at 15,000× *g* for 10 min at 4 °C. Pretests using standard-treated and premium-coated MST capillaries (NanoTemper Technologies GmbH, Munich, Germany) were performed as reported elsewhere. [46] In assay buffer (PBS), the protein did not adsorb to standard-treated capillary walls. Therefore, the following experiments were carried out on standard-treated capillaries. In order to determine the best state for MST signal repeatability and the suppression of unspecific adsorption to capillary walls, buffer conditions were then assessed. The assay buffer was used to dilute compound stocks (5 mM) in MST buffer to the greatest solubility concentration (50 µM). In MST tests, 16-fold 1:1 serial dilutions of each compound were added to NT647-HA (20 µL). The reaction solutions were loaded into standard-treated capillaries after 10 min of incubation at rt, and they were subsequently placed in the chip tray of Monolith NT.115 for thermophoresis analysis and the appraisal of K_D_ values. Signals were captured using 10% LED power and high MST power. K_D_ values were calculated from compound concentration-dependent changes in normalized fluorescence (F_norm_) of HA after 21 s of thermophoresis. Each compound was tested in triplicate. Data examined using MO Affinity Analysis software (v2.3, NanoTemper Technologies GmbH, Munich, Germany). Confidence values (±) were indicated next to the K_D_ value for each tested compound.

#### 3.2.2. Surface Plasmon Resonance (SPR)

SPR binding studies were achieved at 25 °C using Biacore T200 (Cytiva, Uppsala, Sweden). His-HA was captured on the surface of the CM5 sensor chip using the anti-histidine antibody provided in the His Capture Kit (Cytiva, Marlborough, MA, USA). The anti-histidine antibody was diluted to 50 µg/mL in the immobilization buffer included in the kit and covalently coupled to Sensor Chip CM5 by standard amine coupling to a level of approximately 11,000 RU. Then, His-HA was injected (21.5 µg/mL 10 mm acetate, pH 4.59) over the anti-histidine antibody surface for 60 s. On the reference surface, no protein was injected. The dissociation was monitored by injecting a running buffer for 600 s. Glycine buffer (10 mM, pH 1.5, 1 min) was injected to regenerate the surface. HBS-P+ buffer was used as the running buffer. After HA immobilization, the buffer was injected over the chip overnight (5 L/min). The association phase was carried out at 25 °C with a flow rate of 30 µL/min using a solution of peptide in HBS-P+ buffer at several concentrations (from 0.32 to 10 µM), and the dissociation phase was carried out with just the buffer. Using BIAevaluation software (v3.1) provided with the Biacore T200 instrument (Cytiva), the equilibrium dissociation constants (K_D_), kinetic dissociation (k_off_), and kinetic association (k_on_) constants were calculated from the sensorgrams by global fitting of a 1:1 binding model.

### 3.3. Biological Procedures

#### 3.3.1. Cells Line and VIRUS

Hemagglutination assays and/or plaque assays were used to determine the viral stocks’ titers in accordance with accepted practices [48,49]. The Madin–Darby canine kidney (MDCK, ATCC, CRL-2936) cell line was utilized. MDCK cells were grown as reported elsewhere [50]. The Brisbane-like influenza A/H1N1 oseltamivir-resistant virus strain A/Parma/24/09 H1N1 was propagated in 80% confluent MDCK cells as previously described, and viral stocks were stored at −80 °C [51].

#### 3.3.2. Cytotoxicity Assay

This assay was carried out on 80% confluent MDCK grown in 96-well tissue culture microplates in accordance with other reports [50]. Two-fold serial dilutions of peptides (starting from 25 μM) were added to the cells and incubated at 37 °C. Light microscopy was used to analyze the cell morphology after 24, 48, and 72 h. Following this, cells were washed in phosphate-buffered saline (PBS, pH 7.4), and 100 µL of 0.5 mg/mL 3-(4,5-dimethylthiazol-2-yl)-2,5-diphenyltetrazolium bromide (MTT) solution (0.5 mg/mL in PBS) was put to each well. After incubation at 37 °C for 3 h, the MTT solution was discarded and 100 μL of dimethyl sulfoxide (DMSO) were added to each well. Cells were incubated at room temperature for 15 min, and an ELISA plate reader (PerkinElmer Italia, Monza, Italy) with a 570 nm test wavelength and a 690 nm reference wavelength was used to read the microplates. Cytotoxicity was computed from an average of three replicates for each experiment, which was carried out in triplicate. The cytotoxicity was computed by comparing the average OD of wells containing compounds with the average OD of wells containing culture medium alone. Compound dilutions that did not affect cell viability compared to untreated cells were considered non-cytotoxic concentrations and utilized for antiviral assays.

#### 3.3.3. Hemagglutination Inhibition Assay (HI)

First, the A/Parma/24/09 H1N1 virus was titrated using the hemagglutination test, exploiting its ability to agglutinate turkey red blood cells (RBC). To this end, 50 μL of the viral suspension were transferred to the 1st well of 24 of a 96-well U-bottom plate, and then 50 μL of PBS were added from well 2 up to well 24. Two-fold serial dilutions of the viral suspension (contained in the 1st well) were carried out in PBS from the 1st well to the 24th well. Then, 50 μL of turkey RBC suspension (0.5% in PBS) were added to each well, and the plate was incubated at room temperature (RT). After 1 h of incubation, the plate was tilted to let non-agglutinated RBC drip from the bottom of the wells. The reciprocal of the highest dilution of the viral suspension still capable of causing agglutination corresponded to the viral titer. This dilution represents 1 virus-agglutinating unit (HAU). The experiment was repeated six times, and the viral titer was obtained from the mean of six replicates.

A/Parma/24/09 H1N1 virus in phosphate-buffered saline (PBS, pH 7.4) was incubated for 1 h at 4 °C with serial dilutions of compounds in PBS. An equal volume of 0.5% turkey erythrocytes was then added and allowed to agglutinate for 60 min at RT. HI titers were expressed as the concentration of the compound producing 50% inhibition of hemagglutination by four virus-agglutinating units (HAUs).

#### 3.3.4. Neutralization Assay (NT)

Cells were placed into wells of 96-well tissue culture microplates (20,000 cells/well) and incubated for 24 h at 37 °C in 5% CO_2_. NT was performed by incubating equal amounts of a viral suspension containing 105 plaque-forming units (p.f.u.) with serial two-fold compound dilutions, beginning at 25 µM, in culture medium for 1 h at 4 °C. A culture medium without compounds was used as a negative control. Cells were infected with 100 μL/well (three independent experiments were performed) of the virus-compound combinations. After 1 h of incubation, cells were washed carefully and incubated for 72 h at 37 °C. The viral cytopathic effect (c.p.e.) was measured by MTT assay as reported elsewhere by our laboratory [51]. Results were expressed as a percentage of c.p.e. inhibition by comparison with untreated control cultures. EC_50_ was defined as the compound concentration that generates 50% of the maximal protection. The percentage of protection was calculated by the equation (A570 compound-virus − A570 virus)/(A570 mock infected cells − A570 virus) × 100%. The data were expressed as the EC_50_ relative to the value observed in the absence of the compound.

Neutralizing titers were expressed as EC_50_, that is the compound concentration providing 50% inhibition of infection.

### 3.4. Computational Studies

The in silico studies were carried out using the Schrodinger Suite, version 2023-1. The structure-based computational studies were performed on the HA homology models already generated and described in the previous work [46].

The studied peptide derivatives were built in Maestro using the Build facility and submitted to LigPrep calculations to obtain the correct protonation state at physiological pH. Prepared molecules were submitted to minimization using MacroModel, the OPLS4 force field, the GB/SA water solvation model, and the PRCG algorithm to a convergence of 0.01 kJÅ^−1^ mol^−1^.

The SiteMap-calculated molecular interaction fields were used to define the Glide Grid for each HA in the Receptor Binding Site. Docking calculations were carried out using the Glide SP-peptide protocol and saving 100 poses for each ligand. Obtained poses were clustered using a Complete Linkage algorithm, defining the correct number of clusters suggested by the Kelley penalty evaluation. The most populated clusters and best docked compounds were selected for the following analysis.

## 4. Conclusions

In this paper, we synthesized a library of peptidomimetics, N-methyl peptides and peptoids, starting from the most promising tetrapeptides previously identified [23]. Direct binding assays were carried out using two biophysical techniques (MST and SPR). Both demonstrated specific binding between compounds **4**, **9**, **16**, and **18** and HA. Therefore, the antiviral activity of these compounds was assessed through biological assays, such as HI and NT. Results from the hemagglutination inhibition assay confirmed the specific interaction with HA. The neutralization test showed that S(N-Me)LDC (**4**) and SKHNhS (**18**) are the most active derivatives. Computational analysis allowed us to envision the putative interactions of these ligands with HA, where they establish conserved interactions with residues of the RBS, as already observed for peptides **1** and **2** [23]. All applied methods agreed upon the identification of an N-methyl peptide, S(N-Me)LDC, able to bind HA with high affinity and inhibit influenza virus hemagglutination and cell infection at nano- and pico-molar concentrations, respectively. This small sequence can represent a valuable starting point for the design of small molecules. The work carried out opens the way to new perspectives for the development of new anti-influenza drugs, especially in a context in which the emergence of new and drug-resistant viruses highlights the need for new antiviral approaches and strategies.

## Figures and Tables

**Table 1:** Sequence and binding affinities between HA and peptides **1**, **2**, **3**–**18** determined using MST and SPR. The binding affinities of peptides **1** and **2** are reported as references. ^a^ Scala et al. (2017) [23]. All peptides are amidated and acetylated. ^b^ NPD: nonpertinent data. ^c^ Abbreviations of peptoid monomers: *N*hS: *N*-(2-hydroxyethyl)glycine, *N*L: *N-*(isobutyl)glycine, *N*D: *N*-(1-carboxymethyl)glycine, *N*hC: *N*-(2-tioethyl)glycine, *N*K: *N*-(4-aminobutyl)glycine, *N*H: *N*-(1-imidazomethyl)glycine.

Pep	Sequence	MST K_D_ (µM)	SPR K_D_ (µM)
**1** ^a^	SLDC	10.4 ± 0.23	7.12 ± 0.26
**2** ^a^	SKHS	7.26 ± 0.06	4.53 ± 0.08
**3**	(N-Me)SLDC	24.8 ± 0.71	7.60 ± 2.69
**4**	S(N-Me)LDC	0.0325 ± 0.002	0.58 ± 0.34
**5**	SL(N-Me)DC	8.8 ± 0.23	NPD ^b^
**6**	SLD(N-Me)C	0.142 ± 0.03	3.80 ± 1.06
**7**	(N-Me)SKHS	0.336 ± 0.02	4.19 ± 0.86
**8**	S(N-Me)KHS	7.93 ± 0.73	8.33 ± 1.73
**9**	SK(N-Me)HS	0.0528 ± 0.005	1.26 ± 0.36
**10**	SKH(N-Me)S	3.04 ± 0.85	NPD ^b^
**11**	*N*hSLDC ^c^	0.0672 ± 0.002	6.55 ± 1.52
**12**	S*N*LDC ^c^	0.701 ± 0.03	3.81 ± 2.13
**13**	SL*N*DC ^c^	1.34 ± 0.02	NPD ^b^
**14**	SLD*N*hC ^c^	0.0459 ± 0.01	NPD ^b^
**15**	*N*hSKHS ^c^	7.4 ± 0.35	2.09 ± 2.41
**16**	S*N*KHS ^c^	0.0391 ± 0.003	1.70 ± 0.13
**17**	SK*N*HS ^c^	3.07 ± 0.79	NPD ^b^
**18**	SKH*N*hS ^c^	0.0491 ± 0.001	0.43 ± 0.21

**Table 2:** Sequence and HI activity of peptides **1**, **2**, **4**, **9**, **16**, and **18**. The activity of peptides **1** and **2** is reported as a reference [23].

Pep	Sequence	IC_50_ ^b^ (nM)
		A/Parma/24/09 H1N1
**1** ^a^	SLDC	6 ± 3
**2** ^a^	SKHS	1.5 ± 0.7
**4**	S(N-Me)LDC	4.6 ± 2.3
**9**	SK(N-Me)HS	3.2 ± 1.6
**16**	S*N*KHS	1.2 ± 0.6
**18**	SKH*N*hS	3.3 ±1.7

^a^ Scala et al. (2017) [23]. All peptides are amidated and acetylated. ^b^ IC_50_ corresponds to the peptide concentration that inhibits virus-induced hemagglutination by 50%.

**Table 3:** Sequence and Nt activity of peptides and peptoids.

Pep	Sequence	EC_50_ ^a^ (µM)	SI ^b^
**1** ^c^	SLDC	4.6 ± 0.05 × 10^−6^	>5.4 × 10^6^
**2** ^c^	SKHS	4.8 ± 0.12 × 10^−8^	>5.2 × 10^8^
**4**	S(N-Me)LDC	8.5 ± 0.04 × 10^−5^	>2.9 × 10^5^
**9**	SK(N-Me)HS	1.425 ± 0.02 × 10^−4^	>1.78 × 10^3^
**16**	S*N*KHS	4.7 ± 0.02 × 10^−4^	>5.3 × 10^4^
**18**	SKH*N*hS	5 ± 0.04 × 10^−5^	>5 × 10^5^

^a^ EC_50_: the peptide or peptoid concentration that reduced cytopathic effect by 50%; ^b^ SI: the ratio of CC_50_ (the peptide or peptoid concentration that reduced cell proliferation by 50%, equivalent to a concentration > 25 µM) and EC_50_; the standard errors and means from three different experiments are displayed. The mean values of three independent experiments with standard errors are shown. ^c^ Scala et al. (2017) [23].

**Table 4:** Best docking score (glidescore) obtained for studied ligands and 6′-sialyl-N-acetyllactosamine as a reference (see Figure S19 in Supplementary Materials).

Pep	Docking Score Kcal/mol
**1**	−6.760 ^a^
**2**	−6.520 ^a^
**4**	−5.881
**9**	−5.972
**16**	−6.328
**18**	−6.788
**6′-sialyl-N-acetyllactosamine**	−6597

^a^ Docking score values refer to the docking calculations performed in a previous article [46].

## Data Availability

Data are within the article and the Supplementary Materials.

## Supplementary Materials

The supporting information can be downloaded at: https://www.mdpi.com/article/10.3390/ijms241814268/s1.
