# Supplementary material for: Rational Design of Novel Peptidomimetics against Influenza A Virus: Biological and Computational Studies
Source: Int J Mol Sci. 2023 Sep 19;24(18):14268. doi: 10.3390/ijms241814268 (PMC10531517; doi:10.3390/ijms241814268)
Supplement: Supplementary file 1 [file ijms-24-14268-s001.zip › ijms-2554182-supplementary.pdf]

SUPPLEMENTARY MATERIALS

Rational design of novel peptidomimetics against Influenza A virus: biological and computational studies

Maria Carmina Scala [1], Magda Marchetti [2], Fabiana Superti [2,3], Mariangela Agamennone [4], Pietro Campiglia [1] and Marina Sala [1,*]

[1] Department of Pharmacy, University of Salerno, Via Giovanni Paolo II 132, 84084 Fisciano, Italy; mscala@unisa.it (M.C.S.); pcampiglia@unisa.it (P.C.)

[2] National Centre for Innovative Technologies in Public Health, National Institute of Health, Viale Regina Elena, 299, 00161 Rome, Italy; magda.marchetti@iss.it (M.M.); fabiana.superti@iss.it (F.S.)

[3] Association for Research on Integrative Oncology Therapies (ARTOI) Foundation, Via Ludovico Micara, 73, 00165 Rome, Italy; fabiana.superti@artoi.it (F.S.)

[4] Department of Pharmacy, University "G. d'Annunzio" of Chieti-Pescara, Via dei Vestini 31, 66100 Chieti, Italy; magamennone@unich.it (M.A.)

* Corresponding author: msala@unisa.it; Tel.: +39-(0)-89968148

Table of contents:

Supplementary schemes and figures:

**Table S1:**	Analytical data of peptides **1-18**	S2
**Figure S1-S16:**	HRMS spectra and HPLC chromatograms of peptide **3-18**	S3
**Figure S17**	Docked poses of compound **1** in the RBS of studied HAs	S19
**Figure S18**	Docked poses of compound **6** in the RBS of studied HAs	S19
**Figure S19**	Docked poses of 6'-sialyl-N-acetyllactosamine superimposed to the X-ray geometry	S20

Table S1. Analytical data of peptides **3-18**.

**Pep.**	**Sequence**	**HPLC k'[a]**	**HRMS**
**1**[b]	SLDC	3.93	477.18970
**2**[b]	SKHS	4.68	499.26235
**3**	(N-Me)SLDC	8.00	492.2860
**4**	S(N-Me)LDC	9.33	492.2727
**5**	SL(N-Me)DC	5.20	493.9463
**6**	SLD(N-Me)C	10.50	492.9463
**7**	(N-Me)SKHS	7.88	513.2344
**8**	S(N-Me)KHS	0.44	513.2260
**9**	SK(N-Me)HS	0.50	513.2026
**10**	SKH(N-Me)S	0.58	513.2427
**11**	*N*hSLDC	4.55	492.2759
**12**	S*N*LDC	4.75	478.1846
**13**	SL*N*DC	1.15	478.3001
**14**	SLD*N*hC	1.10	493.2882
**15**	*N*hSKHS	1.15	513.2335
**16**	S*N*KHS	1.40	499.2022
**17**	SK*N*HS	2.40	498.9463
**18**	SKH*N*hS	1.20	513.2522

[a] k'=[(peptide retention time-solvent retention time)/solvent retention time]; [b] Scala et al. (2017).

Supplementary figures of Mass spectrometry and HPLC of peptides used in the study

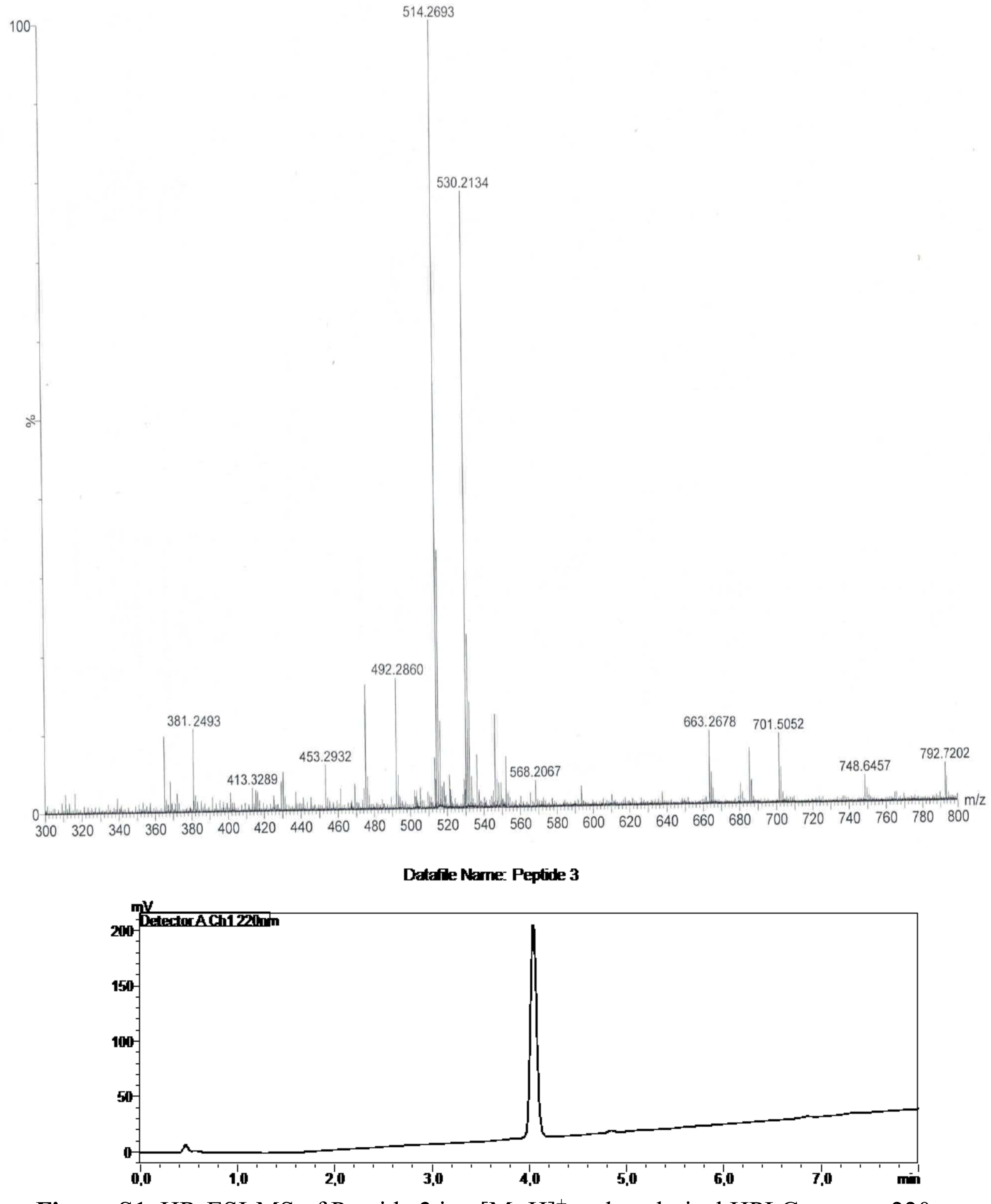

Figure S1. HR-ESI-MS of Peptide **3** ion $[M+H]^+$ and analytical HPLC trace at 220 nm.

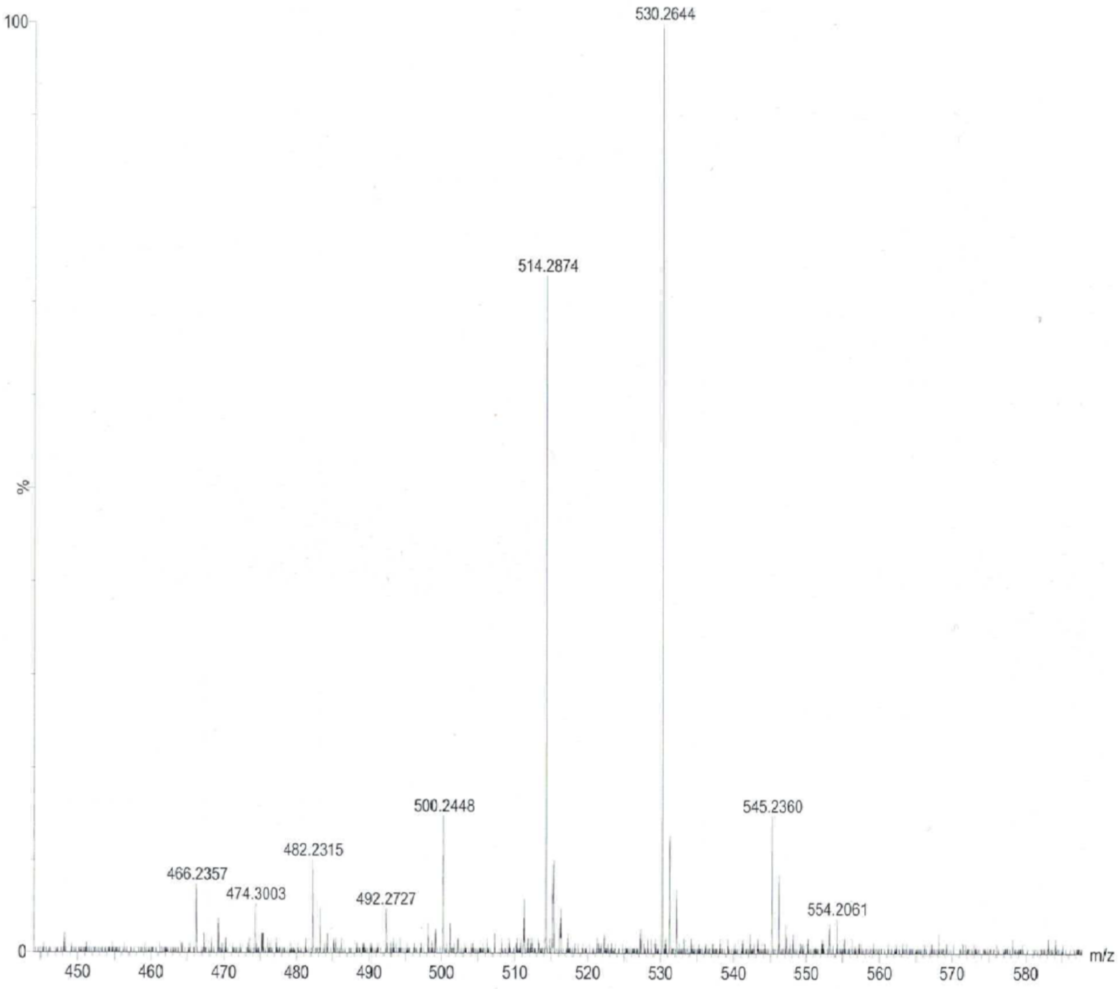

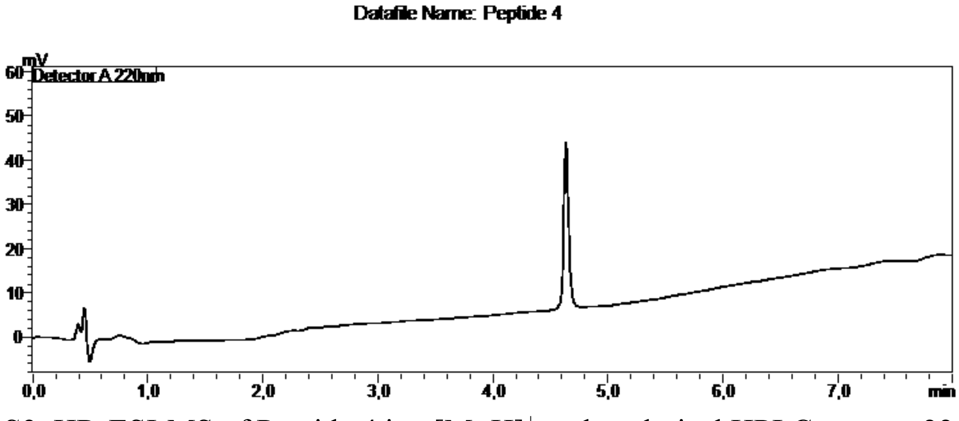

Figure S2. HR-ESI-MS of Peptide **4** ion $[M+H]^+$ and analytical HPLC trace at 220 nm.

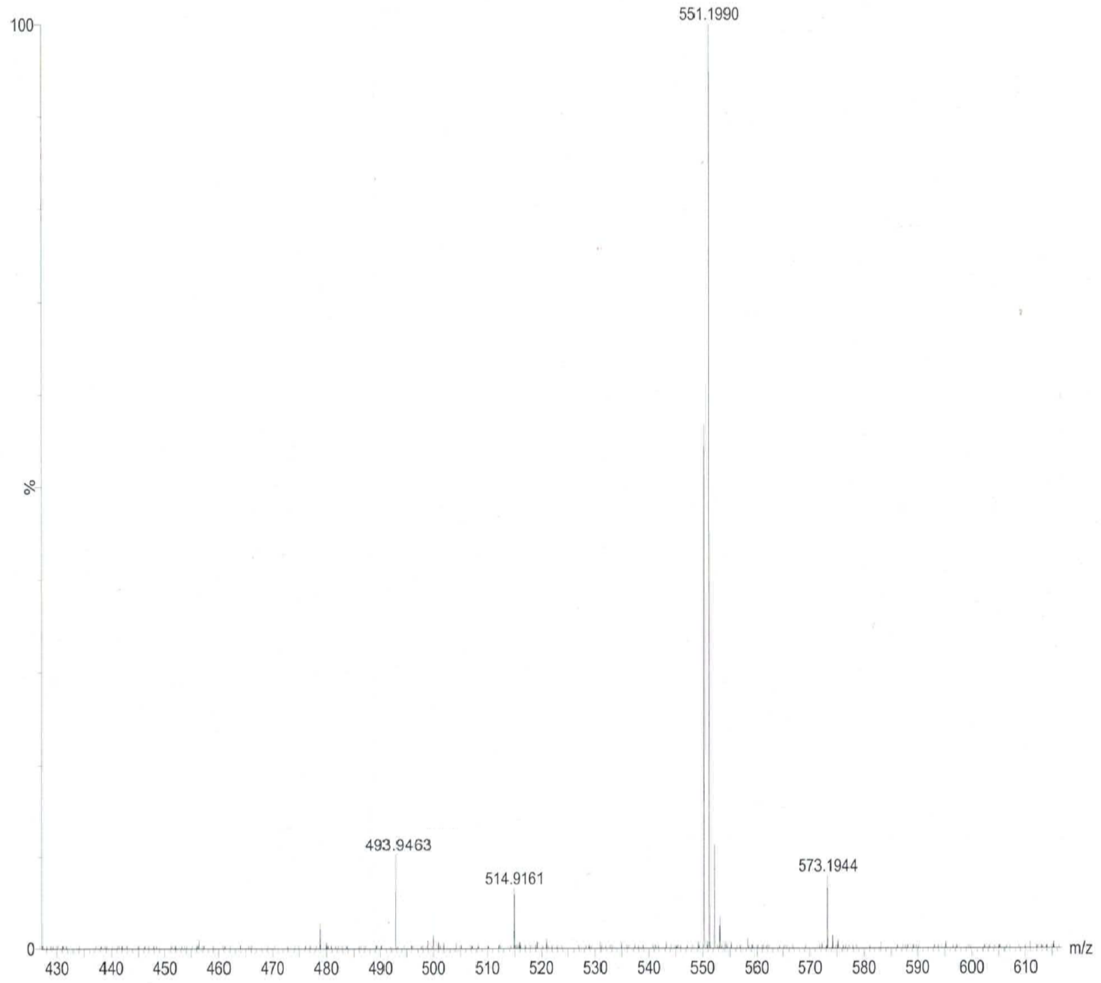

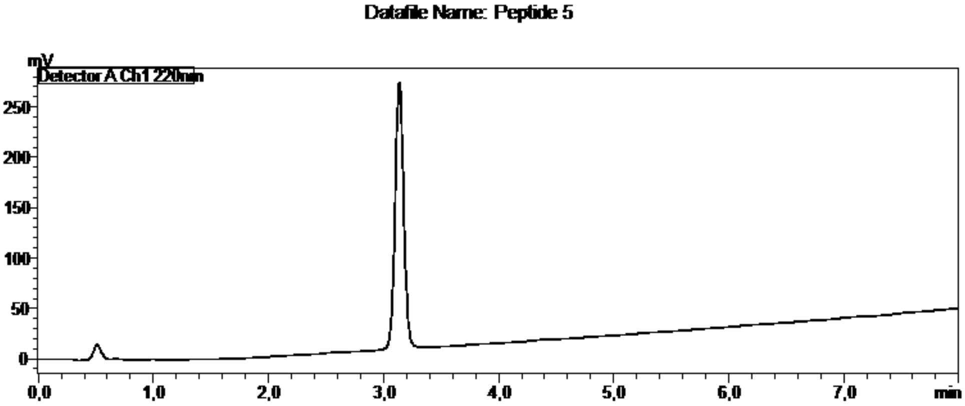

Figure S3. HR-ESI-MS of Peptide **5** ion $[M+H]^+$ and analytical HPLC trace at 220 nm.

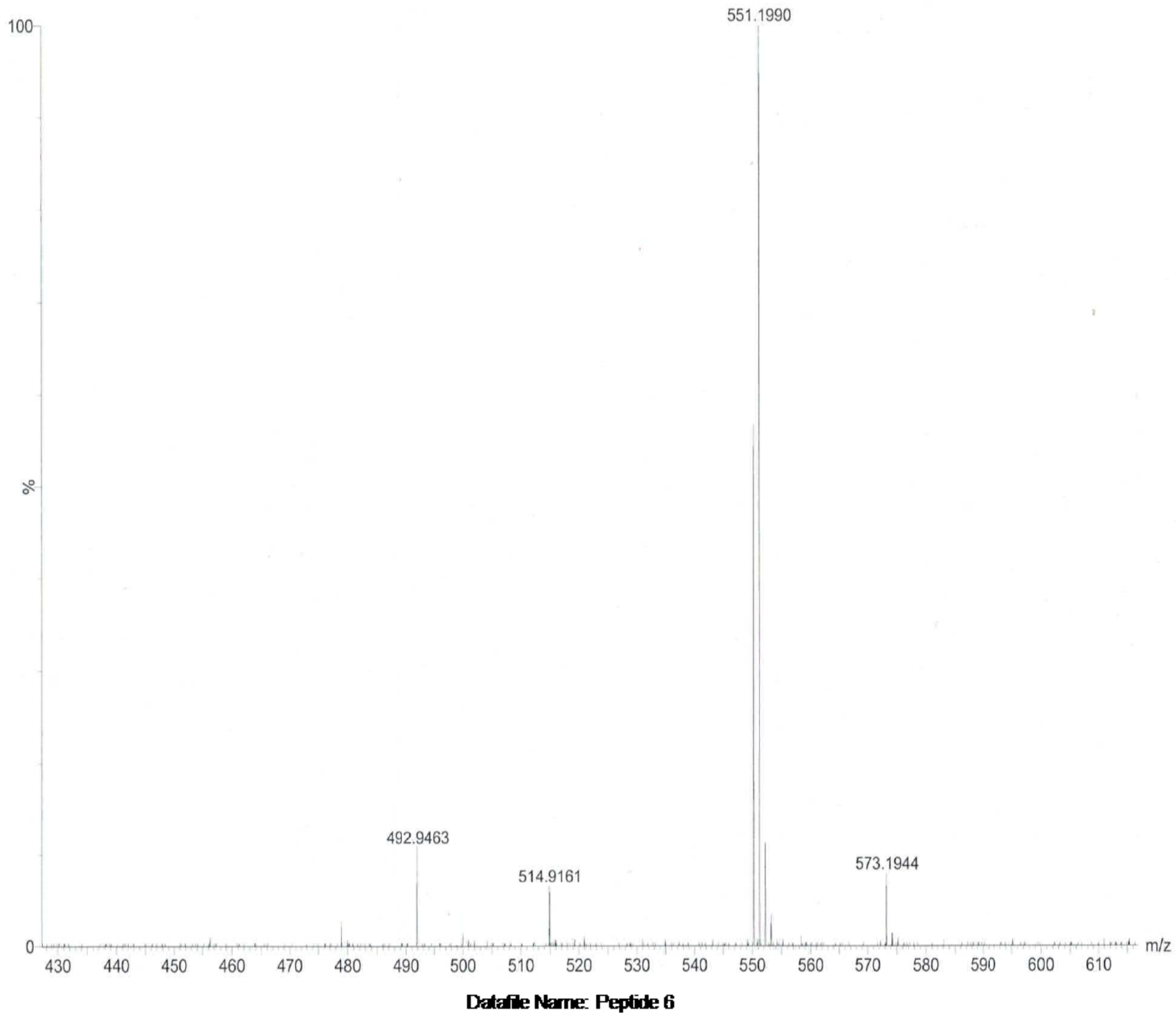

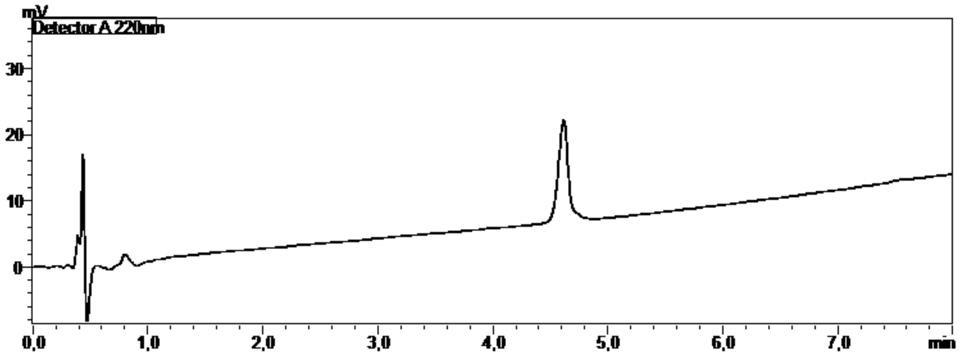

Figure S4. HR-ESI-MS of Peptide **6** ion $[M+H]^+$ and analytical HPLC trace at 220 nm.

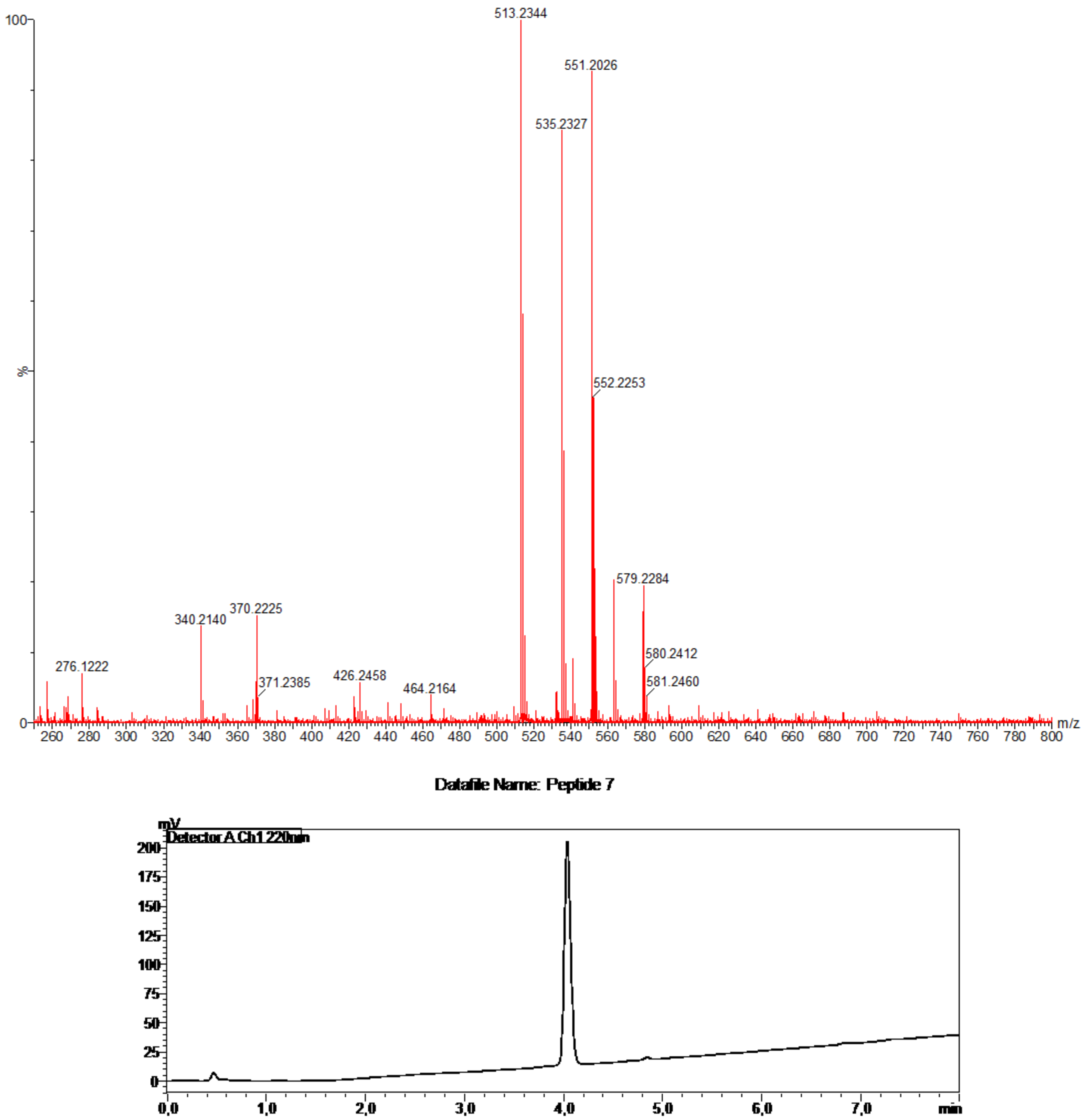

Figure S5. HR-ESI-MS of Peptide **7** ion $[M+H]^+$ and analytical HPLC trace at 220 nm.

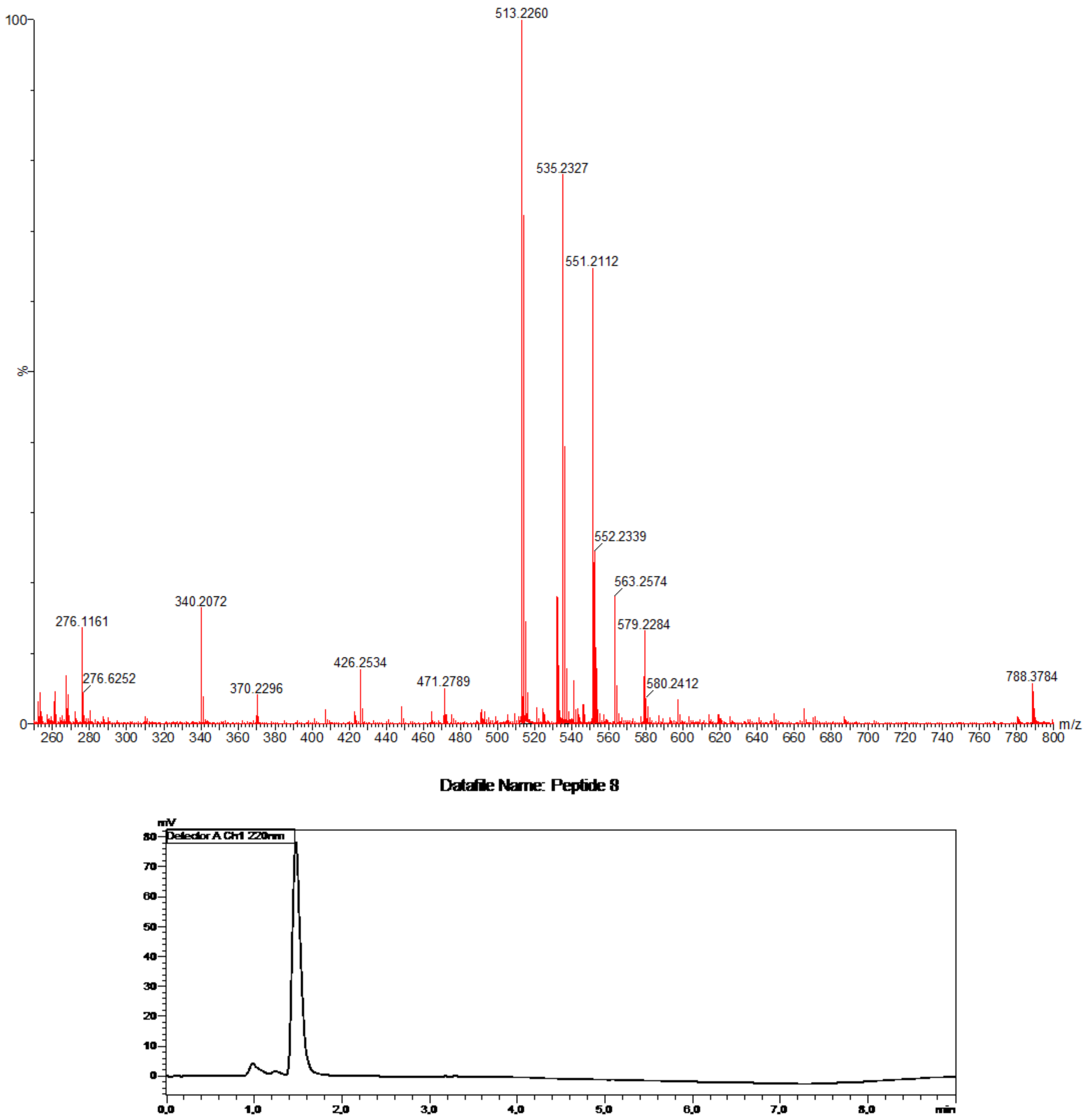

Figure S6. HR-ESI-MS of Peptide **8** ion $[M+H]^+$ and analytical HPLC trace at 220 nm.

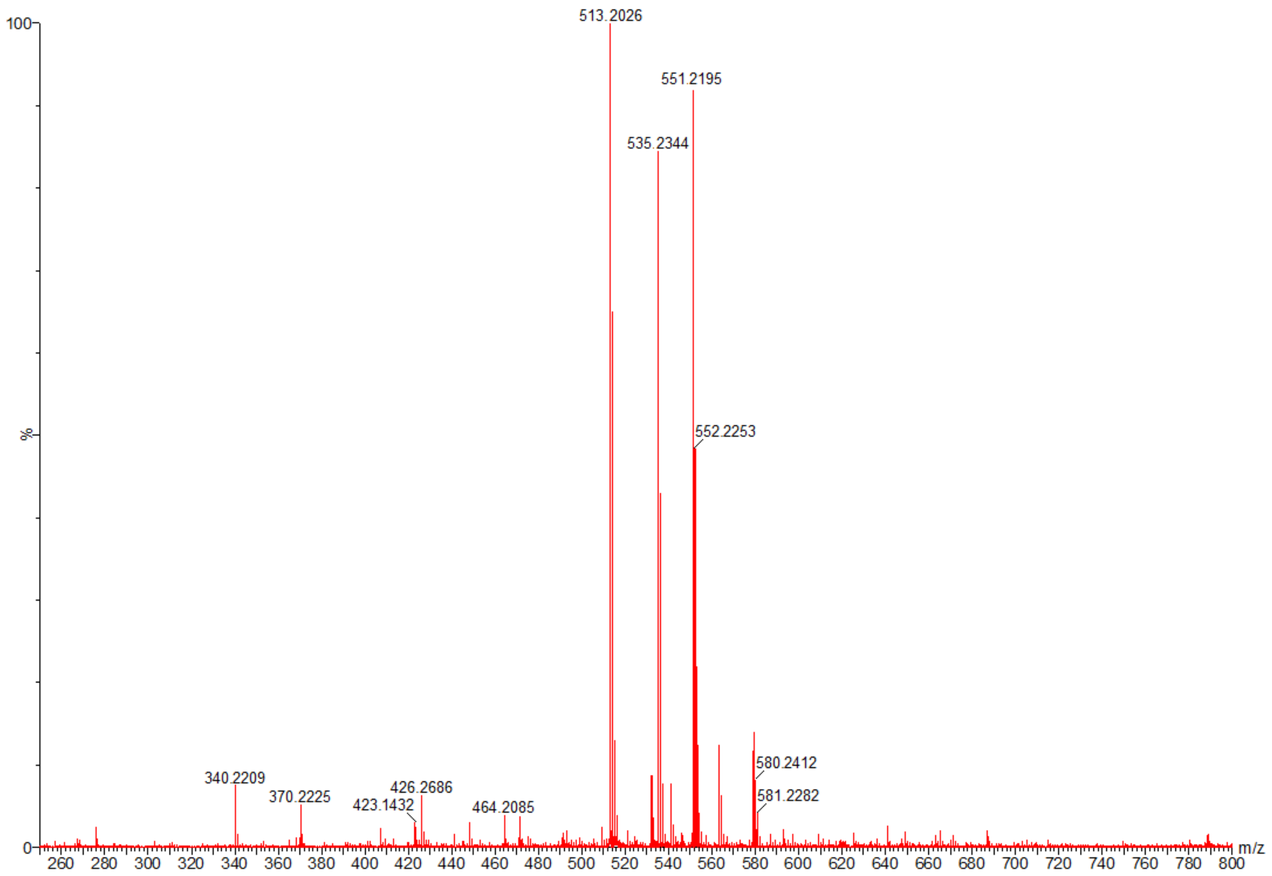

Datafile Name: Peptide 9

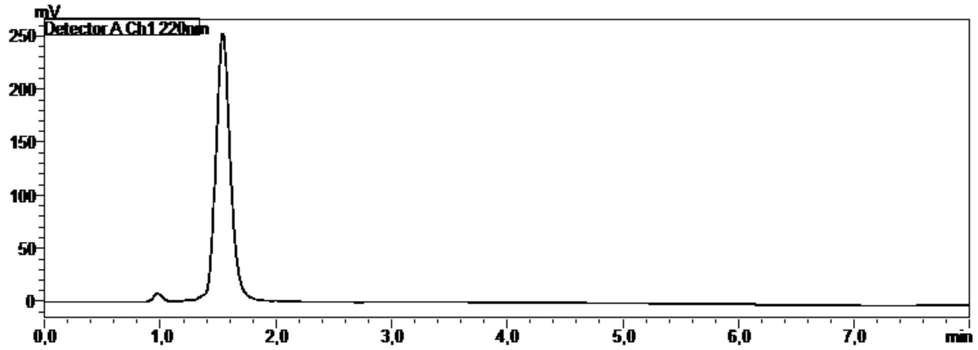

Figure S7. HR-ESI-MS of Peptide **9** ion $[M+H]^+$ and analytical HPLC trace at 220 nm.

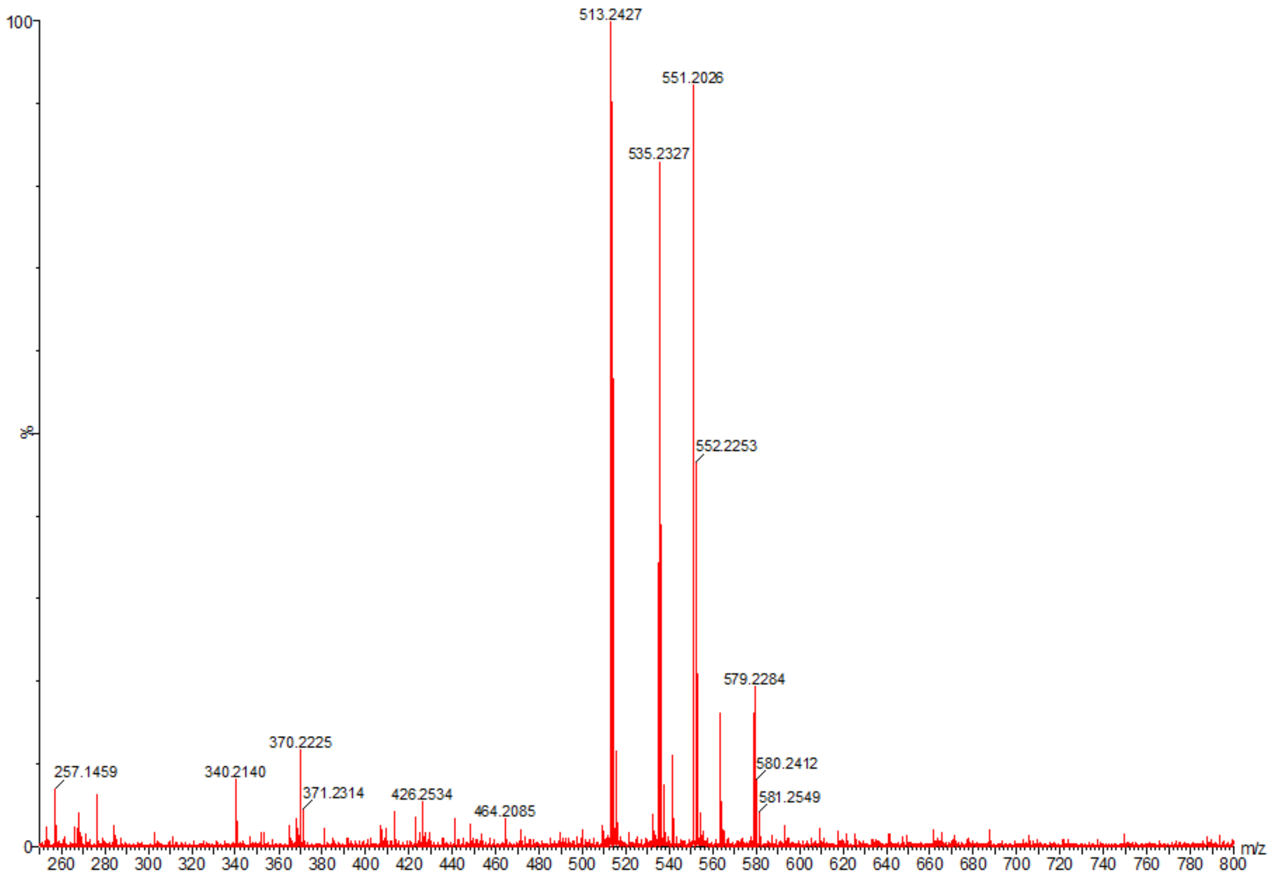

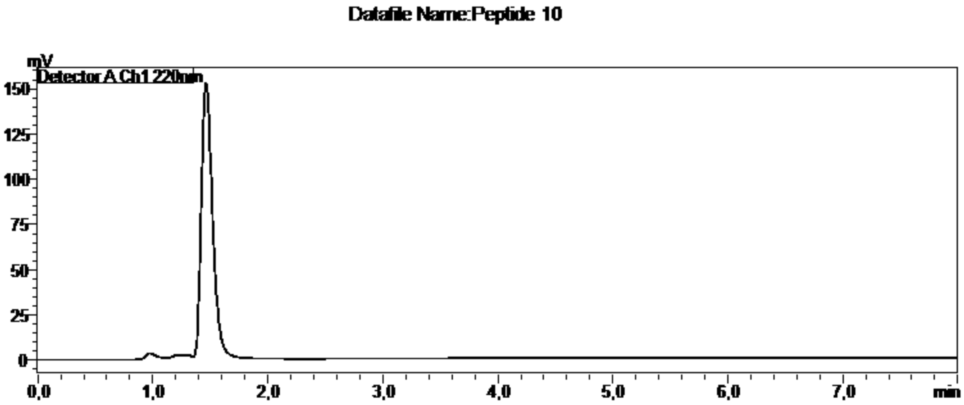

Figure S8. HR-ESI-MS of Peptide **10** ion $[M+H]^+$ and analytical HPLC trace at 220 nm.

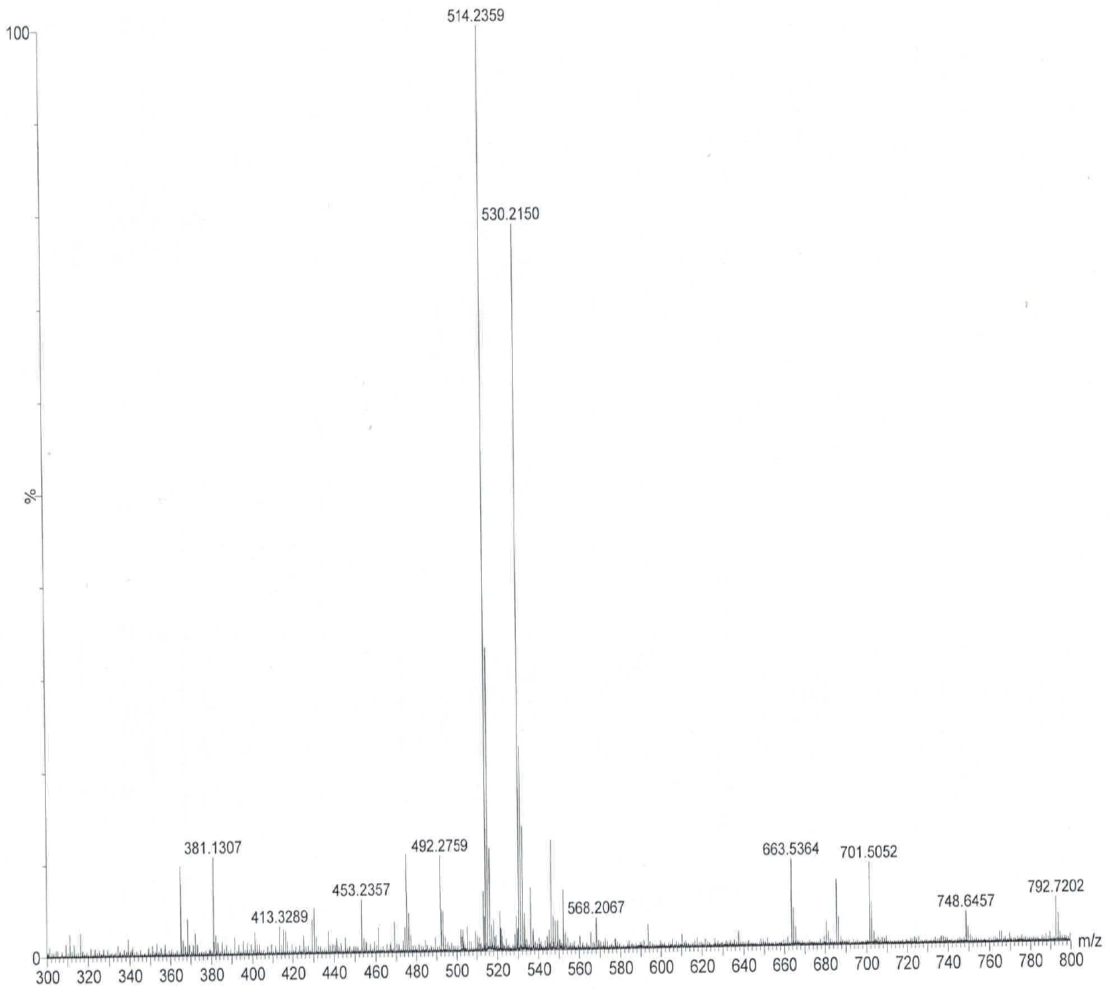

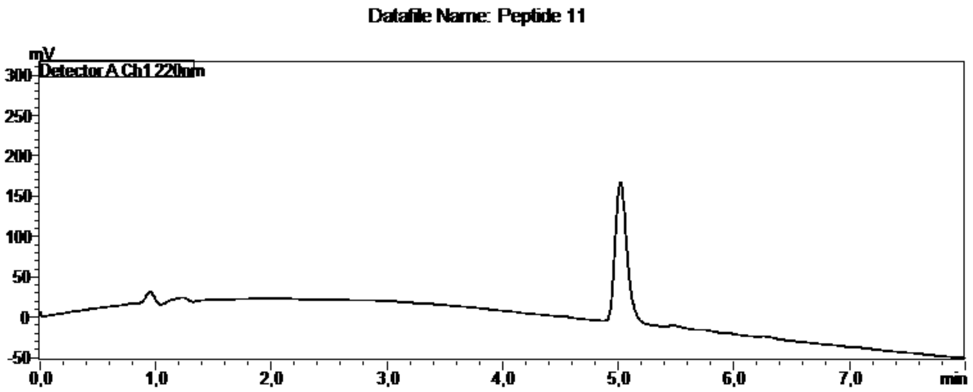

Figure S9. HR-ESI-MS of Peptide **11** ion $[M+H]^+$ and analytical HPLC trace at 220 nm.

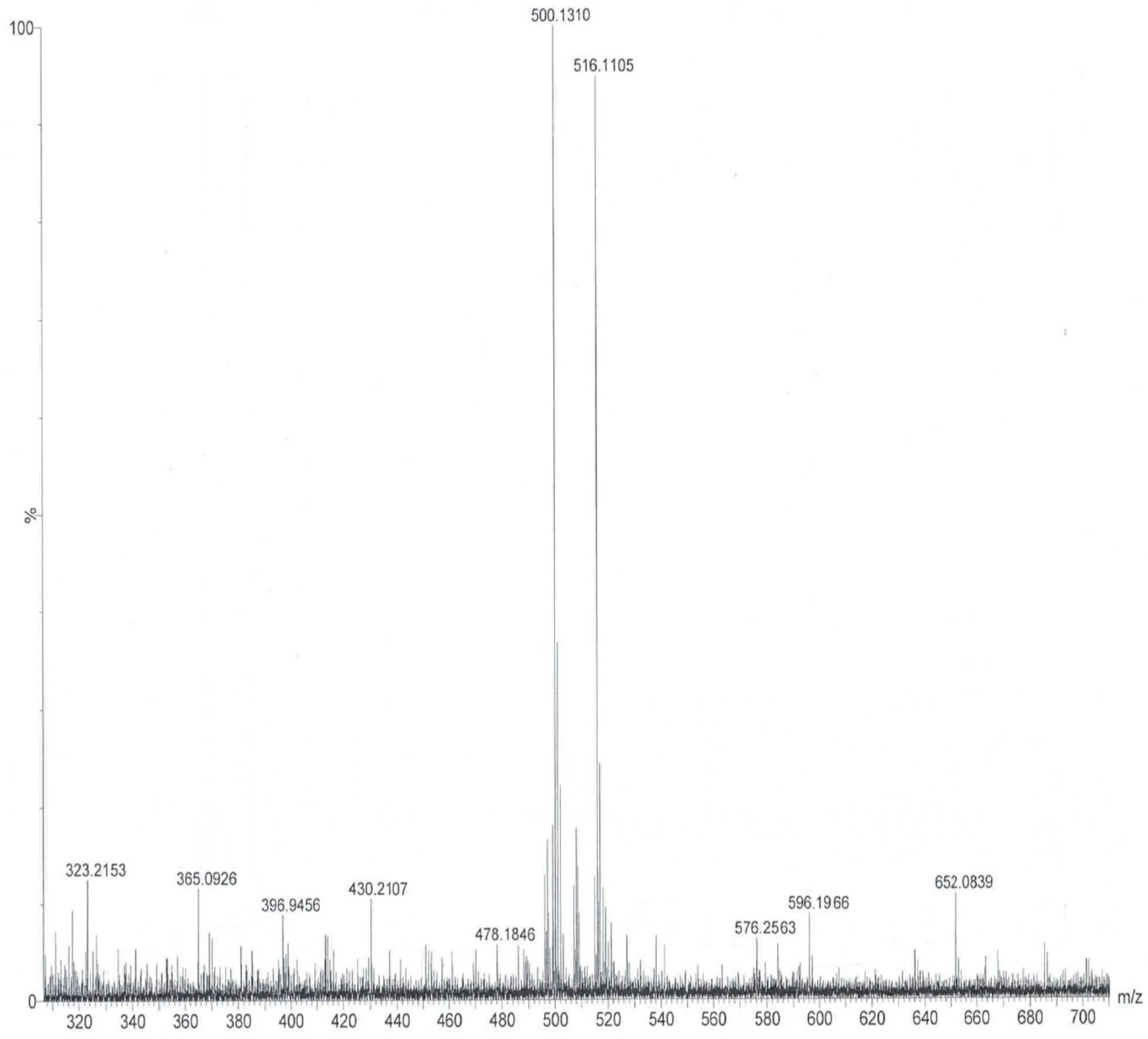

Datafile Name: Peptide 12

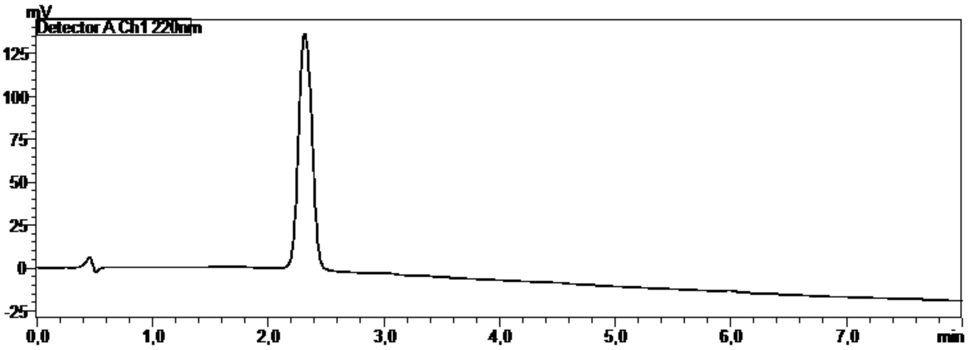

Figure S10. HR-ESI-MS of Peptide **12** ion $[M+H]^+$ and analytical HPLC trace at 220 nm.

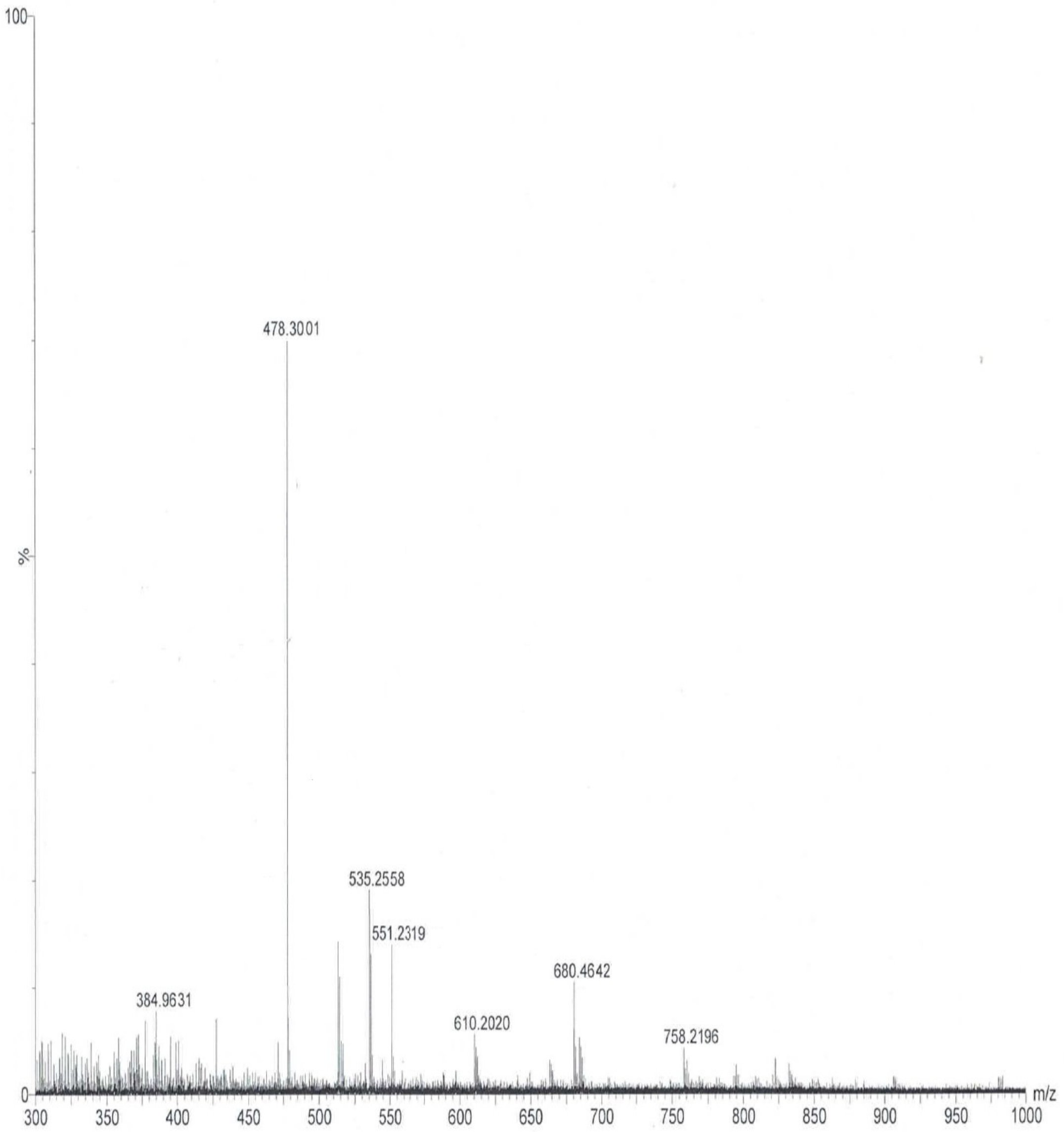

Datafile Name: Peptide 13

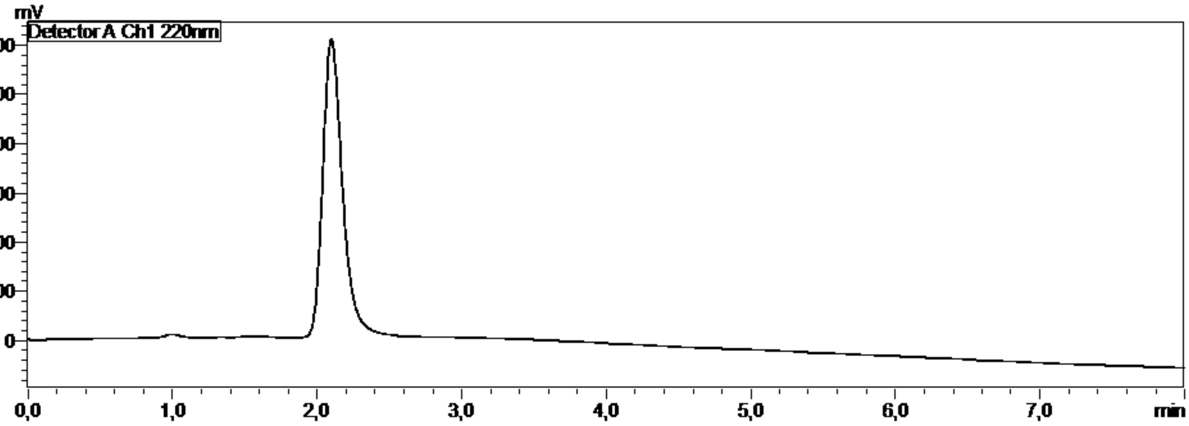

Figure S11. HR-ESI-MS of Peptide **13** ion $[M+H]^+$ and analytical HPLC trace at 220 nm.

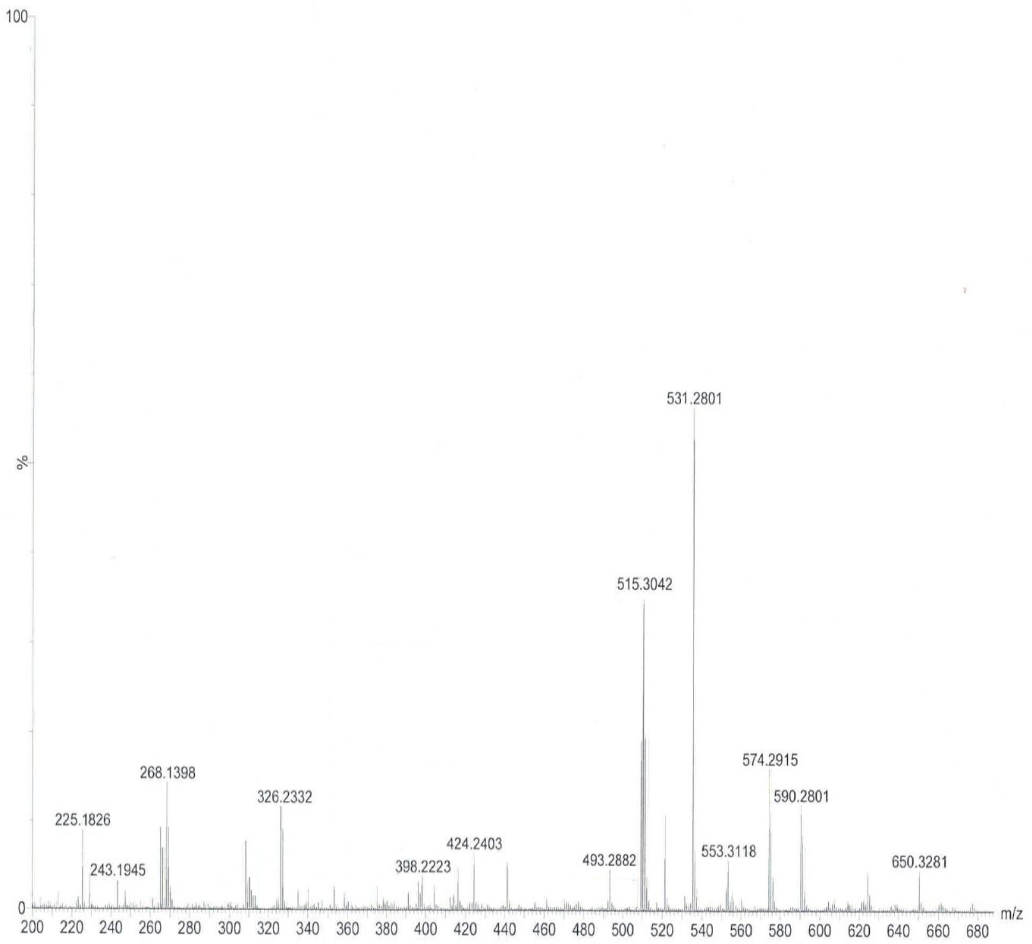

Datafile Name: Peptide 14

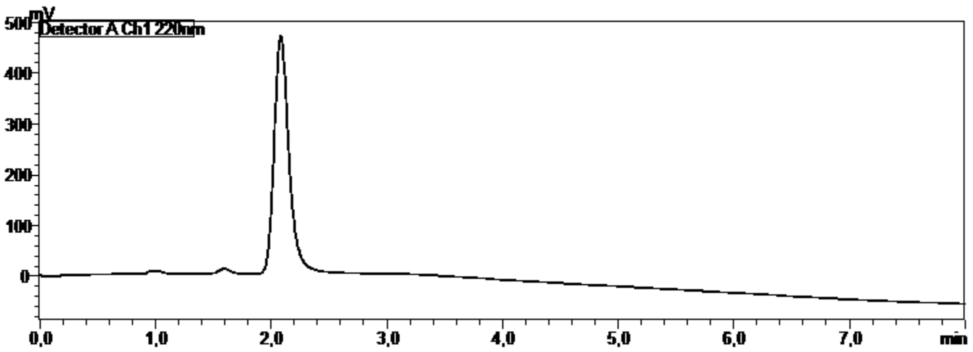

Figure S12. HR-ESI-MS of Peptide **14** ion $[M+H]^+$ and analytical HPLC trace at 220 nm.

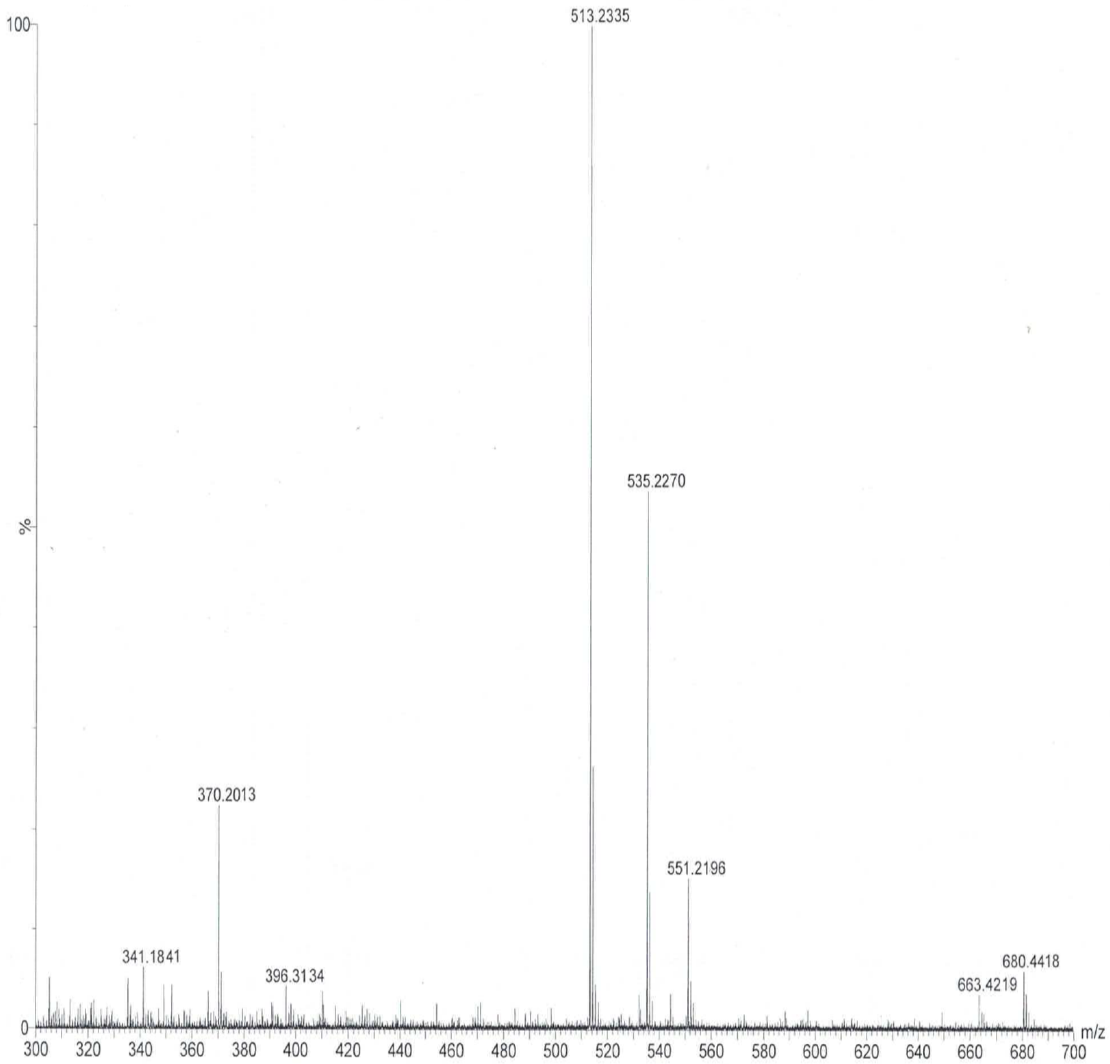

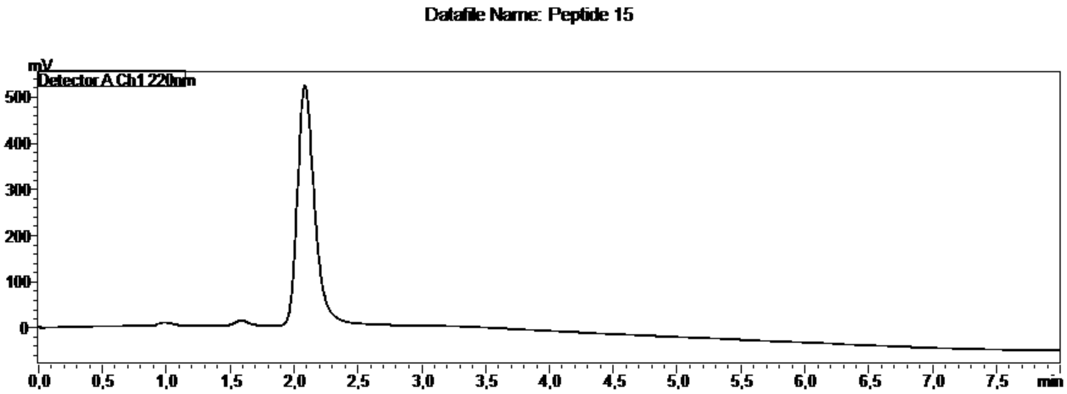

Figure S13. HR-ESI-MS of Peptide **15** ion $[M+H]^+$ and analytical HPLC trace at 220 nm.

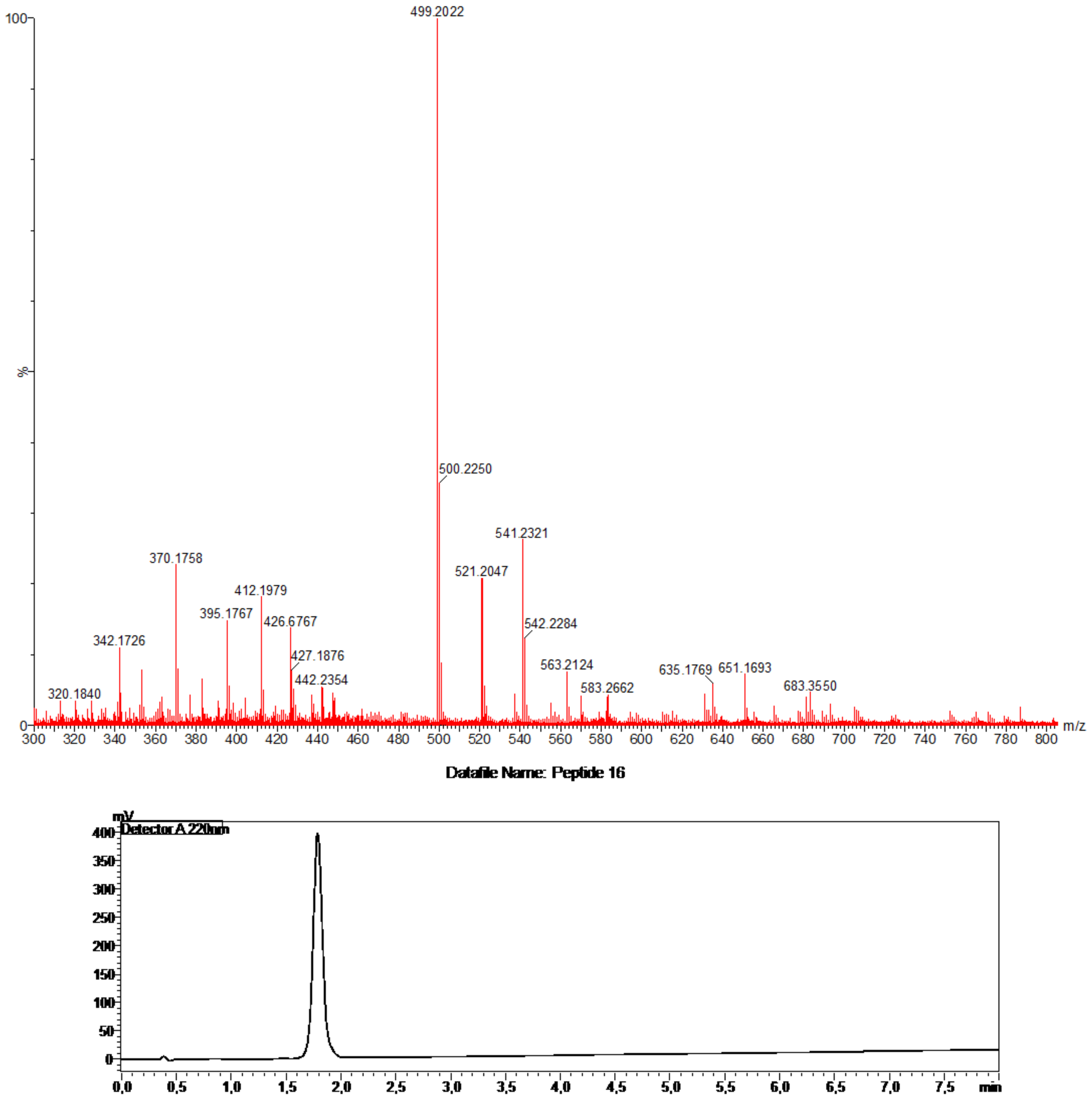

Figure S14. HR-ESI-MS of Peptide **16** ion $[M+H]^+$ and analytical HPLC trace at 220 nm.

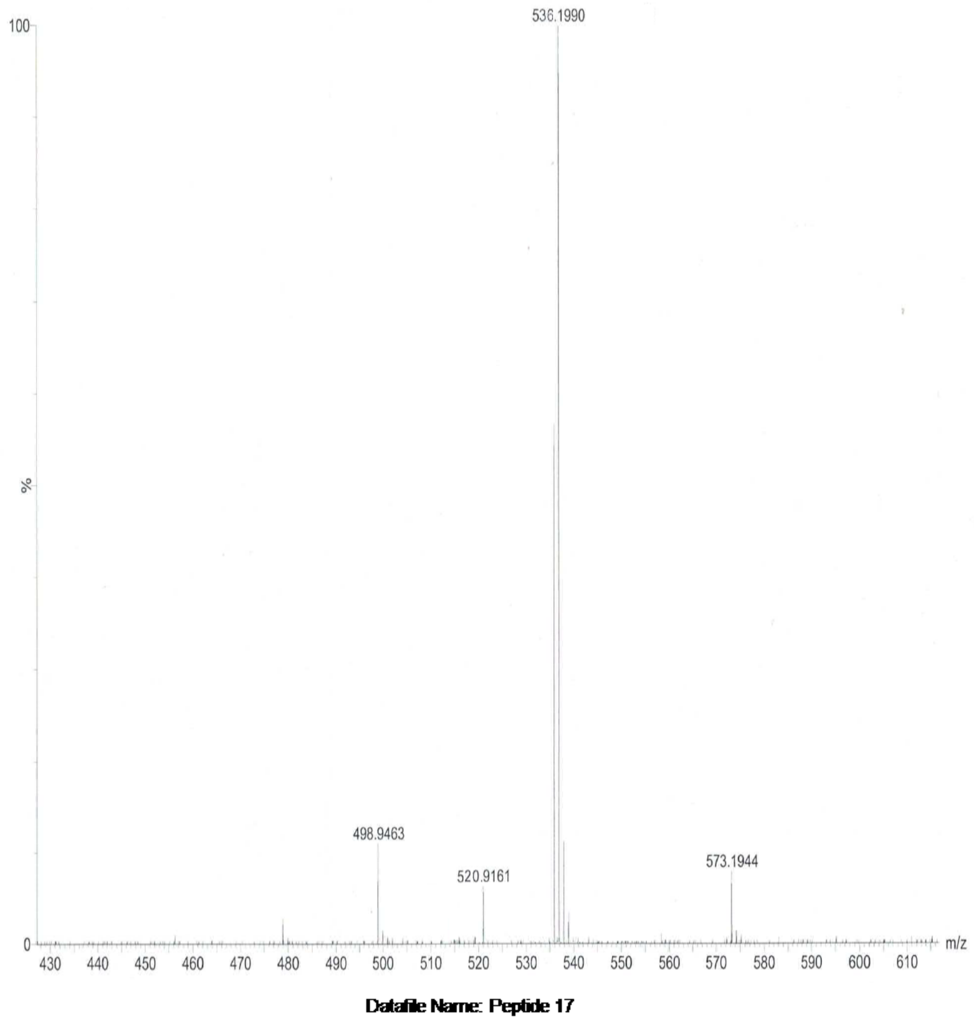

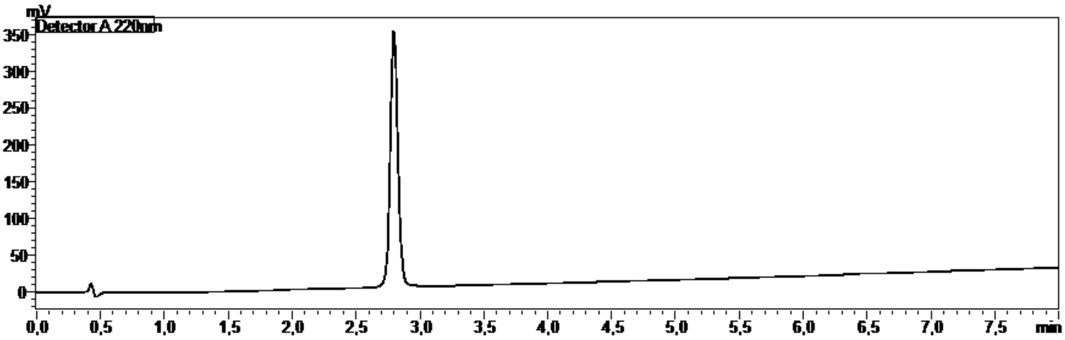

Figure S15. HR-ESI-MS of Peptide **17** ion $[M+H]^+$ and analytical HPLC trace at 220 nm.

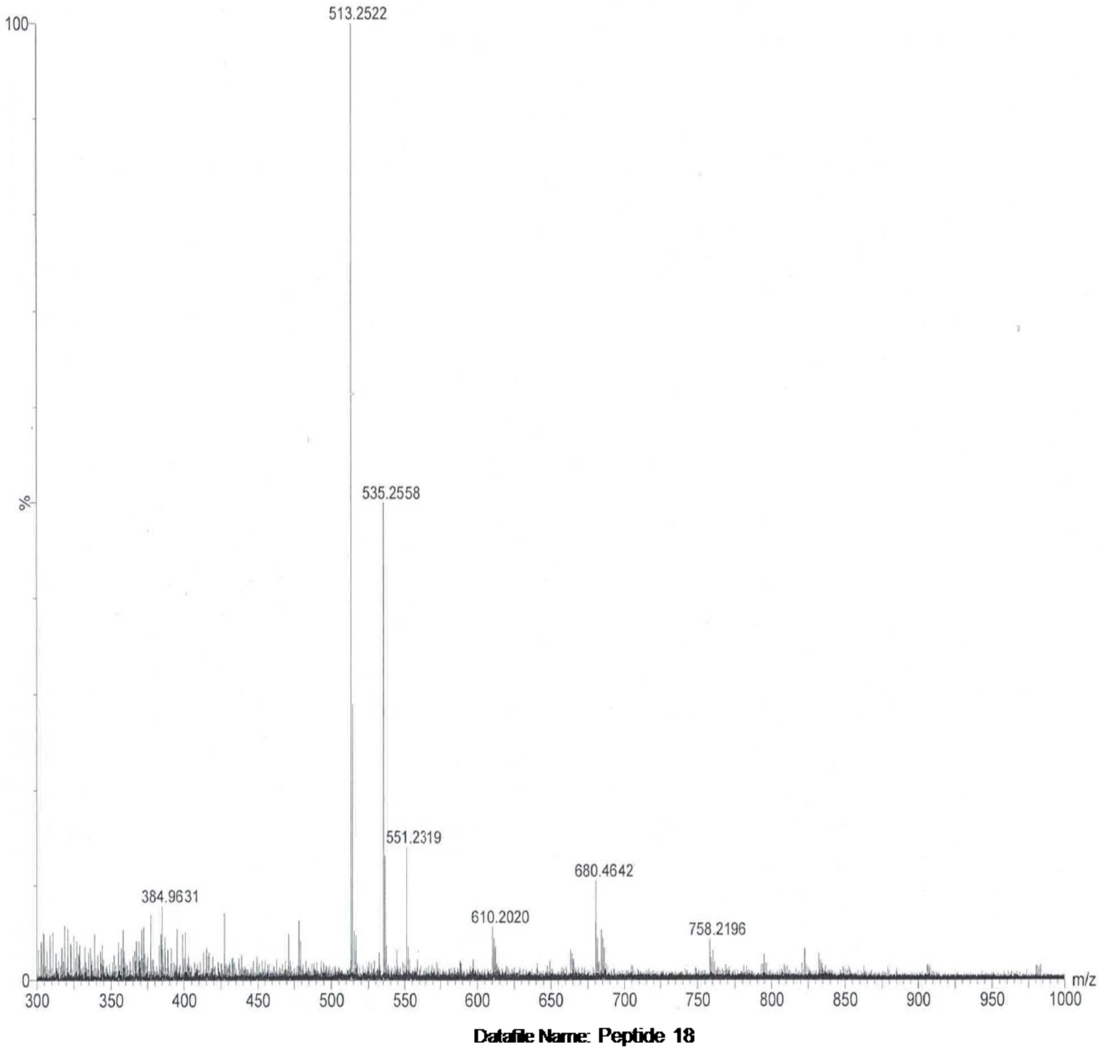

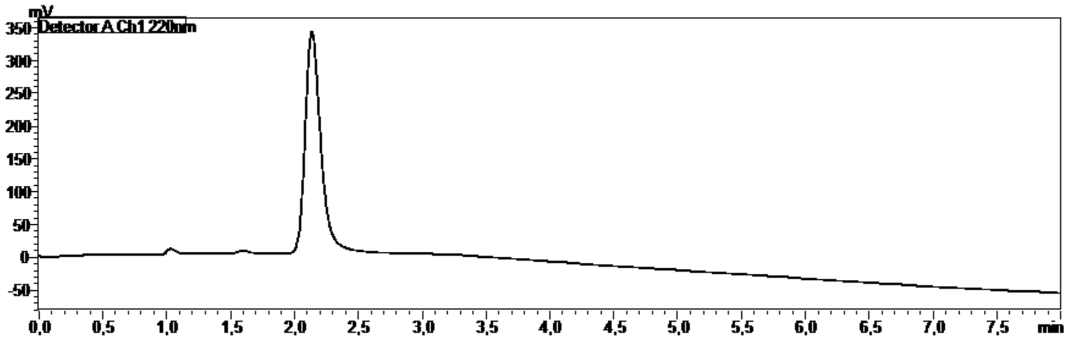

Figure S16. HR-ESI-MS of Peptide **18** ion $[M+H]^+$ and analytical HPLC trace at 220 nm.

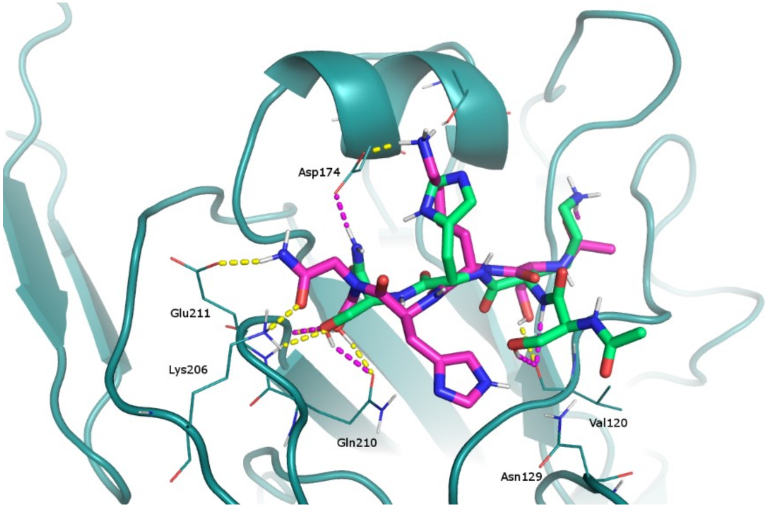

Figure S17. Superimposition of the docked poses of peptoid **18** and reference tetrapeptide **2** represented as sticks (magenta and green C atoms, respectively) in the RBS of A/Parma/24/09 H1N1 (deep-cyan cartoon). Just the HA residues that interact with the ligands are shown as lines for clarity's sake. H-bonds are represented as yellow dashed lines for ligand **18** and magenta for ligand **2**.

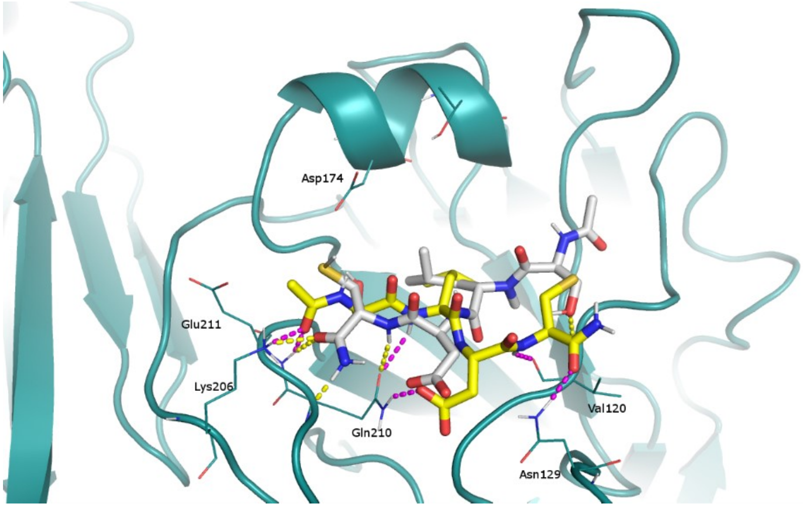

Figure S18. Superimposition of the docked poses of N-methyl peptides **4** and reference tetrapeptide **1** represented as sticks (white and yellow C atoms, respectively) in the RBS of A/Parma/24/09 H1N1 (deep-cyan cartoon). Just the HA residues that interact with the ligands are shown as lines for clarity's sake. H-bonds are represented as yellow dashed lines for ligand **4** and magenta for ligand **1**.

To assess the validity of the docking protocol, the X-ray ligand 6'-sialyl-N-acetyllactosamine was re-docked in the receptor binding site of the H1 HA (PDB ID: 3UBN, Proc. Natl. Acad. Sci. USA **2018**, 115, 4240–4245).
Both Glide SP-peptide and Glide SP docking protocols were applied obtaining comparable results. The RMSD calculated superimposing the crystallographic and docked poses are 0.86 and 0.81, and obtained docking score values are -6.597 and -6.673 with Glide SP-peptide and Glide SP, respectively. (Figure S19)

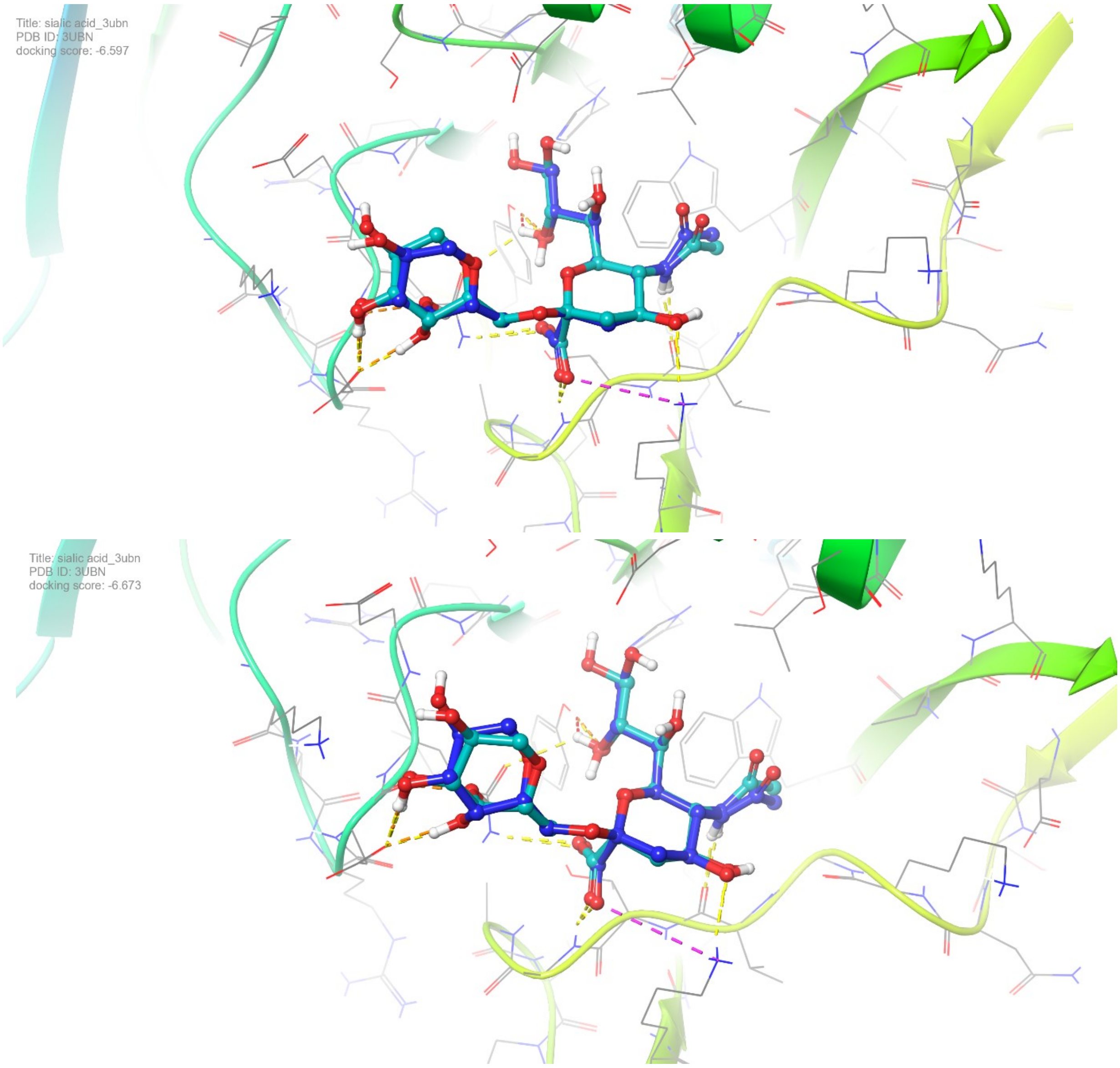

Figure S19. 6'-sialyl-N-acetyllactosamine in its X-ray geometry (blue C atoms), superimposed to its best docked pose (cyan C atoms) obtained with the Glide SP-peptide (panel A) and Glide SP docking protocol (panel B). H-bonds and ionic contacts are represented respectively as yellow and purple dashed lines; the docking score value is reported in the high left corner.
